# Gene expression dynamics before and after zygotic gene activation in *Drosophila* early embryogenesis

**DOI:** 10.1016/j.isci.2025.113272

**Published:** 2025-08-07

**Authors:** Yongwoo Na, Yeon Choi, Thi Thanh My Nguyen, Hoang-Anh Pham-Bui, Jeesoo Kim, V. Narry Kim, Mihye Lee, Jong-Seo Kim

**Affiliations:** 1 School of Biological Sciences, Seoul National University, Seoul 08826, Korea; 2 Center for RNA Research, Institute for Basic Science, Seoul 08826, Korea; 3 Soonchunhyang Institute of Medi-bio Science, Soonchunhyang University, Cheonan 31151, Korea; 4 Department of Integrated Biomedical Science, Soonchunhyang University, Cheonan 31151, Korea

**Keywords:** Natural sciences, Biological sciences, Developmental biology, Transcriptomics

## Abstract

Post-transcriptional gene regulatory mechanisms are fundamental to the determination of gene expression dynamics and especially crucial for the earliest stages of animal development in which transcription is nearly silent. Here, we performed high-resolution total RNA sequencing and quantitative mass spectrometry analysis simultaneously on Drosophila maternal-to-zygotic transition (MZT). Further, this study is the first to report the proteome-wide quantitative changes in protein ubiquitination in *Drosophila* MZT. Our results indicate that timely ubiquitination of the distinct target proteins during MZT is essential for the downregulation of protein expression levels. Profiling of the RNA-associated proteome changes in *Drosophila* MZT suggested that RNA binding can be regulated without the respective change in net protein expression levels for over 200 proteins, including Pcid2, Sym, and Cpsf73. We report that highly dynamic and post transcriptionally regulated protein expression level changes can occur at the earliest stages of the *Drosophila* MZT.

## Highlights

• Proteome and transcriptome expression level dynamics with unprecedented time resolution in MZT
• Ubiquitome profiling in early development and associated degradation of the modified proteins
• Post transcriptional gene regulation and the MZT stage specific RNA-protein interactions

## Graphical abstract

**Figure:**
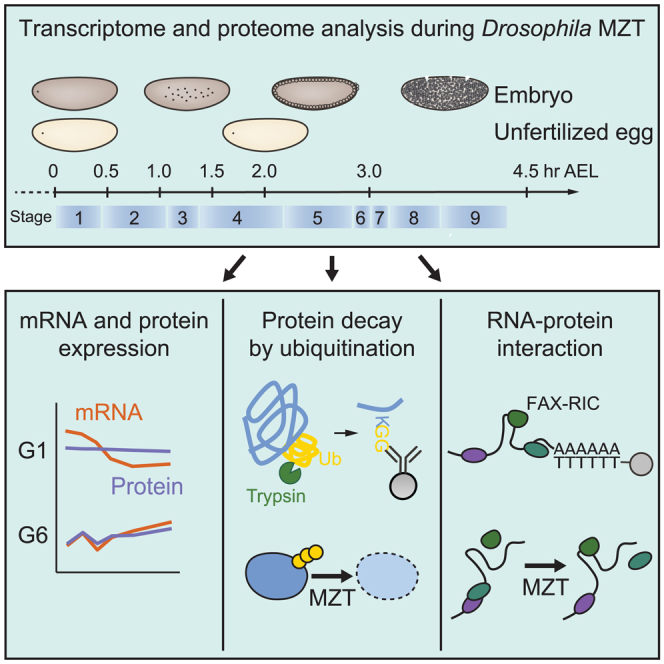

## Introduction

Gene expression is systematically regulated by a series of linked processes including transcription, RNA processing, translation, and decay. The expression of each gene is determined based on the concordant balance in this cascade.^1^ On the transcript level, transcriptional controls primarily determine the amount of gene transcripts to be provided in a given system.^2^ Upon transcription, messenger RNA (mRNA) transcripts are bound with the complex of RNA binding proteins (RBPs) and thereby exist as ribonucleoprotein (RNP) complexes.^3^ Dynamic remodeling of such RNP complexes thereby dictate a myriad of downstream processes which determine not only the character but also timing and the amount of protein expressed in a respective system.^3^ Existence of the splicing system in the nucleus of the eukaryotic organisms allow the single mRNA transcript to be processed differently and code for multiple forms of the protein products.^4^ On the other hand, translational control and transcript stability regulatory mechanisms in the cytoplasm determine the amount of proteins to be produced from each transcript based on the rate of protein synthesis and the duration of each transcript to be available for the translation processes, respectively.^5^^,^^6^^,^^7^^,^^8^ Existence of such multilayered post-transcriptional regulatory mechanisms allow the biological system to both maintain and rapidly alter net protein abundance.^9^ The development of reliable protein quantification techniques based on proteomics analysis has enabled the determination of the genome-wide relationship between transcripts and protein expression.^10^ Initial studies, from those on budding yeast to human cell lines, found a limited correlation between transcript and protein expression, demonstrating that transcript level measurement alone cannot adequately predict or account for protein expression level measurement.^11^^,^^12^^,^^13^ Although the measurement of translational efficiency using techniques such as ribosome profiling was expected to improve the correlation between transcript and protein levels, the correlation between them was still relatively low, indicating that protein expression level is influenced by additional factors beyond the amount of protein produced from each gene transcript.^13^^,^^14^ Results from such studies also indicated that measurement of protein expression level is critical for the proper understanding of gene expression regulatory mechanisms in a given system.

Final layer in the cascade of gene expression control is the stability of each gene’s protein products. While the factors that determine each protein’s stability or half-life are still not completely understood, post translational modification (PTM) of the proteins have been extensively studied in terms of their role in the alteration of the modified proteins’ stability. Among the most abundant and well-studied PTMs are phosphorylation, ubiquitination, acetylation, and methylation.^15^^,^^16^^,^^17^^,^^18^ Recent development of the proteomics analysis techniques for such PTMs have greatly facilitated our understanding of the role of PTMs in gene expression control.^17^^,^^18^^,^^19^^,^^20^ Unlike the other well-known PTMs, protein ubiquitination involves attachment of a small protein called ubiquitin.^20^ The poly ubiquitination, in which multiple ubiquitin proteins are attached to a single site of target protein through the formation of an ubiquitin chain, is known to promote protein decay through the recruitment of tagged proteins to the proteasomal degradation pathway.^21^ Unique properties of the protein ubiquitination also suggest that multiple strategy for modified protein enrichment can be developed. While antibody based and peptide level enrichment of the di-glycine remnant on tryptic peptides have been widely utilized, new strategies are still actively being developed.^20^^,^^22^ Further development and the application of such methodology will facilitate in-depth analysis of protein ubiquitination and advance the exploration of its physiological relevance in diverse biological contexts. Taken together, previous studies have indicated that our understanding of gene expression dynamics is still far from complete, and the critical factors that influence protein levels have not yet been fully determined.

While gene expression control mechanisms are universally present and essential for the whole process of embryonic development, early embryos undergo dramatic and unique changes in their gene expression landscape, during which oocyte-specific features are eliminated and embryonic features are newly established. Developmental processes are directed by the post-transcriptional control of maternally inherited mRNAs in early embryos immediately after fertilization since the transcriptional input from the newly constituted zygotic genome is largely insignificant. Maternal control mechanisms based on the inherited mRNAs and proteins prepare the embryos to use their own genome and establish zygotic control, which is defined as the maternal-to-zygotic transition (MZT). Therefore, MZT processes that are universally present in eukaryotic organisms require the harmonized coordination of post-transcriptional control of maternal mRNAs and transcriptional control of zygotic mRNAs.^23^^,^^24^^,^^25^ For decades, numerous studies have precisely elucidated the mechanisms governing the fate of maternal transcripts and birth of zygotic transcripts, focusing on certain individual mRNAs that play critical roles in the MZT.^26^^,^^27^ Recent studies also elucidated unique and critical roles of post transcriptional gene regulation related to RNA modifications, cytoplasmic poly adenylation and deadenylation of poly-A-tail, and codon optimality in MZT.^28^^,^^29^^,^^30^^,^^31^ Such findings thereby underscored the importance of intricate post transcriptional regulatory mechanisms in early embryogenesis. Accordingly, protein expression studies in early embryos have also found that measurement of transcript expression level alone cannot adequately represent the landscape of protein expression level in early embryos.^32^^,^^33^^,^^34^

Here, we performed in-depth transcriptome and proteome analyses to determine distinct gene expression changes in *Drosophila* MZT. Our analysis revealed discrete clusters of gene expression changes are also associated with distinct Gene Ontology (GO) terms, indicating that there are coordinated regulatory mechanisms for the functionally related genes. Further analysis comparing changes in gene expression levels and translational efficiency suggested that distinct post-transcriptional regulatory mechanisms achieve increased protein expression before and after zygotic genome activation (ZGA). We also report proteome wide change in the level of protein ubiquitination during the *Drosophila* MZT for the first time and found that most of the protein down regulation occurred along with the relatively high levels of protein ubiquitination. Transcripts bound by key regulatory RNA-binding proteins (RBPs) were enriched in different clusters of gene expression changes, further highlighting the importance of RBPs in shaping the gene expression landscape. Formaldehyde crosslinking-based characterization of RNA interactome changes also revealed altered RNA-binding activities of hundreds of RBPs during the MZT without significant changes in their protein levels. Our results provide quantitative and experimental evidence for the shift in the landscape of the post translational protein modifications and RNA-protein interactions during the MZT and suggest that they are major contributor to the removal of the maternally deposited protein products and zygotic genome activations.

## Results

### Global analysis of mRNA and protein expression changes in *Drosophila* MZT

**Figure 1:**
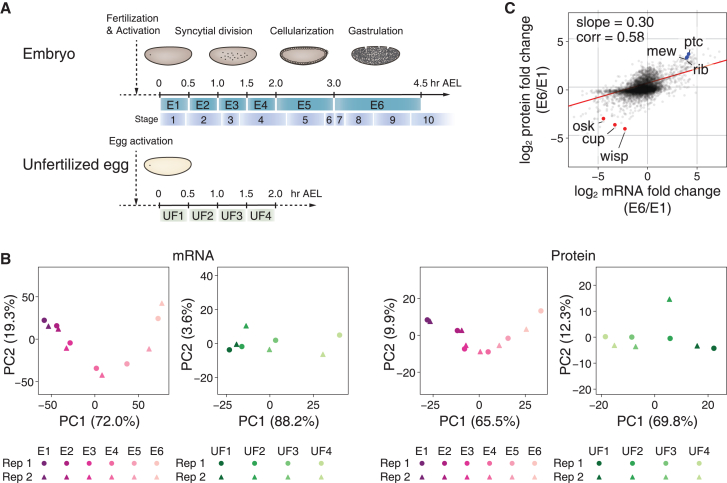
Overview of transcriptome- and proteome-wide analyses in *Drosophila* MZT (A) Schematic outline of the *Drosophila* embryo and unfertilized egg collection time points. The respective developmental stages are noted above the timeline, and distinct time points are represented with E1–E6 and UF1–UF4 for the embryos and egg samples, respectively. (B) Principal component analysis of the transcriptome- and proteome-wide results (*N* = 2; Rep1 and Rep2) at distinct time points. The proportion of variance explained by each principal component (PC) is indicated as percentage in axes labels. (C) Dynamic range of mRNA and protein level changes during 0–4.5 h AEL in *Drosophila* embryogenesis. “Corr” indicates the Pearson correlation coefficient between mRNA and protein fold changes.

We first performed an in-depth gene expression change analysis in *Drosophila* MZT based on the genome wide measurement of both transcript and protein expression. Our analysis was designed to incorporate the embryos in the syncytial division, blastulation, and gastrulation of early *Drosophila* embryogenesis for the precise recording of gene expression dynamics during MZT (Figure 1A). While ZGA was thought to occur following the midblastula transition around 2 h AEL, previous studies have also found significant transcriptional activity at an earlier time point in the MZT.^35^^,^^36^ *Drosophila* eggs can be activated without the event of fertilization and kinetics of maternal transcript regulation is known to be largely indistinguishable from that of the fertilized embryos.^37^^,^^38^ We therefore utilized the activated but unfertilized egg samples at four different time points at 30 min intervals to validate maternally provided post-transcriptional gene regulatory mechanisms, distinguishing them from any zygotic transcription-derived gene expression control in early embryos before the midblastula transition (Figure 1A).

To ensure perfect coordination between our transcript and protein expression levels, a fraction was taken from each of the 10-time point samples and subjected to either RNA-seq or quantitative MS analysis. The samples were then subjected to the next generation sequencing (NGS) for the in-depth measurement of RNA expression levels. For the proteome analysis, peptide samples were labeled with isotopically distinguished 10-plex TMT reagents and SPS-MS3 analysis was utilized to measure the protein expression level changes with high accuracy and reliability.^39^ The use of isobaric tagging also allowed us to perform offline pre-fractionation of the peptide samples to maximize the sensitivity of peptide identification. Such analytical techniques provided highly reproducible measurements of the changes in RNA and protein expression levels that were done in duplicates (Figures S1A and 1B). We then performed the comparison with the recently reported single embryo RNA sequencing data.^40^ Since the reported data consisted of 84 time points spanning a pseudo timescale of 0–3 h, we reconstructed the dataset to align with the time point intervals of our experiment. Upon analysis, we observed a relatively high correlation between the two experiments for both E4 to E1 and E5 to E1 time points. The result further demonstrated the high reproducibility of our transcriptome analysis and accuracy of our sample collection (Figure S1B).

Our data also revealed dramatic changes in both RNA and protein levels during early embryonic development (0–4.5 h AEL) (Figure 1C; Table S1). The protein expression levels of well-known oocyte-specific genes, including *wisp*, *cup*, and *osk*, were downregulated by more than 8-fold, and genes involved in morphogenesis, such as *ptc*, *rib*, and *mew*, were upregulated by greater than 8-fold for both the mRNA and protein expression (Figure 1C; Table S1). Our analysis determined expression level change dynamics of nearly 6000 genes during the MZT, which is a significant expansion from the ∼4000 genes whose gene expression dynamics were measured in the whole span of *Drosophila* embryogenesis.^41^ It is also important to note that the same study determined the gene expression levels at all five time points from 0 h to 4 h, corresponding to our analysis, for less than 2900 genes.^41^ Our analysis thus substantially expanded the repertoire of the genes whose gene expression dynamics are reported in *Drosophila* MZT. Taken together, we report a highly comprehensive analysis of changes in gene expression that covers *Drosophila* MZT.

### Distinct dynamics of gene expression suggest widespread post-transcriptional regulations related to distinct gene functions

**Figure 2:**
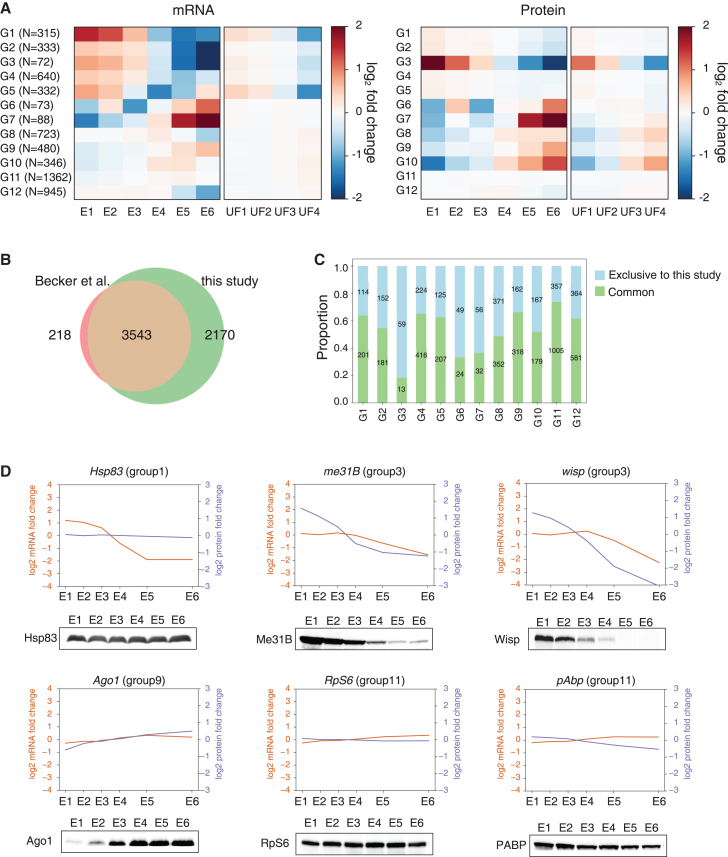
Clustering analysis of mRNA and protein expression levels (A) K-means clustering analysis of the embryonic mRNA and protein expression changes yielded the groups G1–G12, with the number of the genes in each group denoted as N (number). Groups were defined based on combined mRNA and protein expression levels at time points. The color of each cell represents the mean log fold change of mRNA (left panels) and protein (right panels) for the genes at each time point of embryos (E1-E6) and unfertilized eggs (UF1-4). (B) Comparison of the embryonic proteome profile from the previous study by Becker et al.^41^ with our study. (C) The number of identified proteins common to both studies or exclusive to our study is presented for each group of gene expression pattern analysis. (D) Western blot analysis of the embryo samples for the representative genes. Their respective mRNA and protein expression fold-changes are also shown.

As we performed high resolution gene expression analysis in Drosophila MZT, we first asked if we could characterize the distinct pattern of gene expression level changes at each step of MZT that have not been revealed based on the low-resolution analyses. We performed k-means clustering based on changes in both embryonic mRNA and protein expression and categorized the genes into group 1 to 12 (G1-12) (Figure 2A). Clear separation of each gene group based on the pattern of gene expression level changes at individual time point indicate the versatility of both transcripts and protein expression level changes within the developmental stages that last for less than an hour. As aforementioned, our results significantly expanded the number of genes whose transcript and protein expression levels are measured within the context of Drosophila MZT. Compared to the previous study,^41^ which measured the gene expression dynamics across the 0–20 h of embryogenesis with longer and sparser time points of embryo collections, over 2000 genes were unique to our MZT proteome profiling (Figure 2B). Intriguingly, we also found that such genes were overrepresented by the G3, G6, and G7, whose gene expression levels were either greatly up- or down-regulated in our analysis (Figure 2C). We thus report some of the most dynamic changes in gene expression level on both transcript and protein level for the first time in *Drosophila* MZT. Taken together, we report that our MZT centered gene expression level profiling enabled the in-depth and precise measurement of gene expression changes.

We then tried to further characterize the distinct pattern of gene expression changes throughout the MZT. We first found that there are a significant number of genes with strong downregulation in the mRNA expression levels during MZT, belonging to G1–5, G8, and G12. Our detailed gene expression analysis allowed us to distinguish G1-5 from G8 and G12 in which mRNA downregulation was only observed post-ZGA. G1 and G5 genes especially exhibited noticeable changes in RNA expression in unfertilized egg samples, indicating that such processes are based on maternally provided regulatory mechanisms. Intriguingly, while G1 transcript levels remained low from E1 to E6, G5 transcript levels were clearly upregulated from E4 to E6, indicating that substantial zygotic gene transcription replenished the transcript expression of G5 genes (Figures 2A and S2). We then found that despite the strong downregulation in transcript expression levels, protein expression levels were largely unchanged, except for G3 and G8 genes. Among the most notable genes with transcript decay and stable protein expression pattern was *hsp83*. Previous studies have reported that the decay of *hsp83* transcript is based on its association with the RBP, Smg.^42^^,^^43^^,^^44^ However, whether a similar change occurs in protein expression levels remained to be shown. We further validated the stable expression of Hsp83 protein via western blot, demonstrating that a decrease in transcript level does not result in a concomitant decrease in the protein expression level of the *hsp83* gene (Figure 2D). Among the groups of genes with the decreased mRNA expression levels, we observed strong downregulation in protein expression levels of the G3 genes throughout the time point that we analyzed in both embryos and unfertilized eggs (Figures 2A and S2). Our results demonstrate that protein expression downregulation occurs for at least 72 genes in Drosophila MZT and such process is maternally driven and likely activated at the initial phase of embryogenesis. Western blot analysis of the G3 proteins, Me31B and Wisp, further validated our results (Figure 2D). For further validation, we performed western blot analysis using available antibodies and confirmed the relative changes in protein expression levels for Ago1, RpS6, and Pabp. Consistent with the previous reports,^11^^,^^41^ we found a general lack of correlation between transcript and protein expression levels for most of the genes we analyzed with notable exceptions of the G3 and G7 whose value of median correlation coefficient exceeded 0.9 (Figure S3A). Closer examination of the gene expression level change pattern of the G3 genes suggested that transcript expression level downregulation predominantly occurred from E4 to E6, and largely unchanged in unfertilized egg samples, suggesting that transcript decay is based on zygotic genome activation. In contrast, the respective downregulation in protein expression level appeared to be occurring at the beginning of embryogenesis. Taken together, our results suggest that protein downregulation of G3 genes preceded that of the downregulation in transcript expression level indicative of the transcript level independent regulation of the protein expression level. On the other hand, we observed an increase in transcript expression level from E4 to E6, indicative of zygotic genome activation, for G7 genes, resulting in concurrent increase in protein expression level. Moreover, we also observed similarly high correlation for G6 and G9 genes, with median correlation coefficient of 0.88 and 0.86 respectively, whose gene expression levels are also upregulated from E4 to E6. Taken together, our results suggest that unlike most of the changes in transcript expression level during MZT, ZGA results in concurrent changes in total protein expressions (Figures S2 and S3A). It is also interesting to note that both RNA and protein expression levels are especially low at the initial stage for G6 and G7 genes, which show both high correlation and dynamic change in gene expression level. These findings suggest that relatively high levels of existing gene products contributed to the general lack of correlation for the rest of gene expression dynamics (Figure S3B).

**Figure 3:**
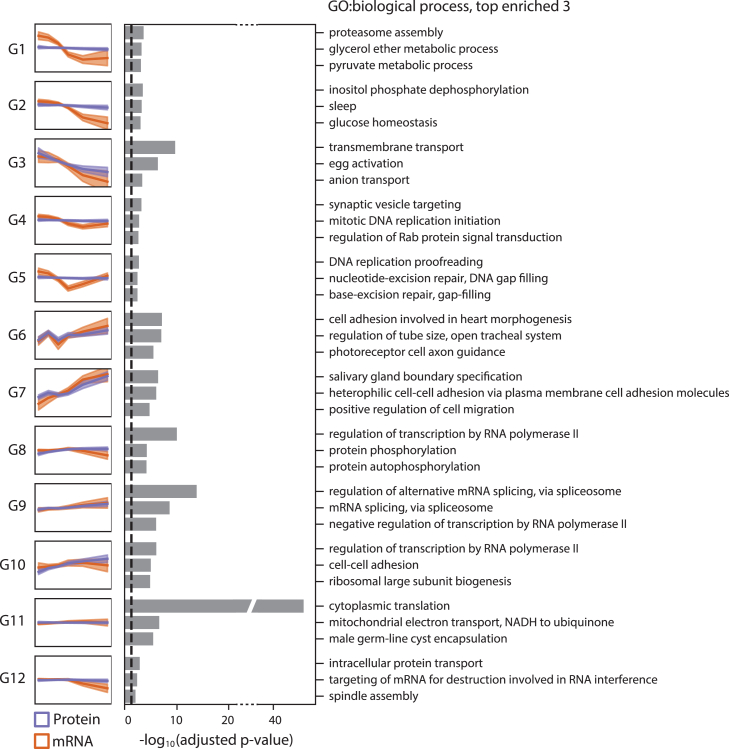
Gene Ontology (GO) terms overrepresented by each group Top three enriched GO terms for each group. The black dashed line indicates an adjusted *p*-value of 0.05. The enrichment *p*-value for each GO term was calculated and adjusted using TopGO. The small line plots on the left indicate the median (dark color) and standard deviation (light color) of RNA and protein temporal dynamics.

Over 1400 genes in our analysis belong to G1, G2, G4, and G5, in which no significant change in protein expression level was observed despite the downregulation in transcript expression level. Intriguingly, further time point specific protein expression level change analysis within the cluster found that there is strong downregulation of the Dhd protein expression level from E1 to E2 time point. Such change was also observed in the unfertilized egg samples at the corresponding time points. We also found similar pattern of changes for four other genes, hts, mute, Nop17l, and CG2201. The rapid decrease in Dhd level immediately after fertilization or egg activation aligns with its role in oocyte to embryo transition.^45^ The result is also consistent with western blot analysis for Dhd protein reported previously.^46^ We thus identified few of the distinct downregulation in protein expression level which may have unknown role in oocyte to embryo transition (Figure S4A). On the other hand, we also found that G1, G2, and G4, G5 can be characterized by their differences in transcript expression post-ZGA. Interestingly, genes belonging to G1 and G2 were enriched in various metabolic processes and proteasome assembly-related protein decay processes. In contrast, G4 and G5, in which transcript levels increased at the last time point, were represented by genes associated with DNA replication processes (Figure 3). These results suggest that there are mRNA expression regulatory mechanisms for functionally related genes, which likely contribute to the stable protein expression of the respective genes. Distinct enrichment of the terms related to egg activation in G3 genes also indicates that *Drosophila* MZT relies on highly selective mechanisms for the removal of proteins that may be unnecessary or hinder subsequent embryogenesis (Figure 3). Enrichment of RNA metabolic processes and transcript processing in the G8, G9, and G10 genes is also consistent with the essential role of such genes in the post-ZGA stages. Genes belonging to G11 and G12 were highly linked to protein translation and transport, indicative of the stable gene expression of these housekeeping genes (Figure 3). On the contrary, genes essential for the differentiation occurring after the blastoderm stage around 3 h AEL belong to G6 and G7, suggesting that they are provided by zygotic gene transcription and underscoring the necessity for precisely timed gene expression for the proper development of *Drosophila* embryos (Figure 3). We then asked if a clear distinction in the gene expression kinetics of G6 and G7 can be explained by the level of pioneering factor zelda binding at the promoter which was reported previously.^47^ Clear enrichment of the genes with high level of Zld biding in G7, and noticeable depletion of such genes in G6, is consistent with the previous finding that Zld binding level is correlated with the earlier transcription of the zygotic genes. The results once again demonstrate the accuracy of temporal dynamics measured in our study. Intriguingly, we also find that there is notable enrichment of high Zld binding genes at G9 and G10. Interestingly, we found that protein expression level of the same group of genes, especially that of G10, are strongly increased while there is relatively small change in the transcript expression level. The results suggest that newly transcribed gene products can drive relatively strong and more efficient increase in the protein expression level. (Figure S4B) Altogether, we report that functionally relevant groups of genes are similarly regulated at both the transcript and protein levels throughout the *Drosophila* MZT.

### Upregulation of protein expression is based on distinct regulatory mechanisms before and after ZGA

**Figure 4:**
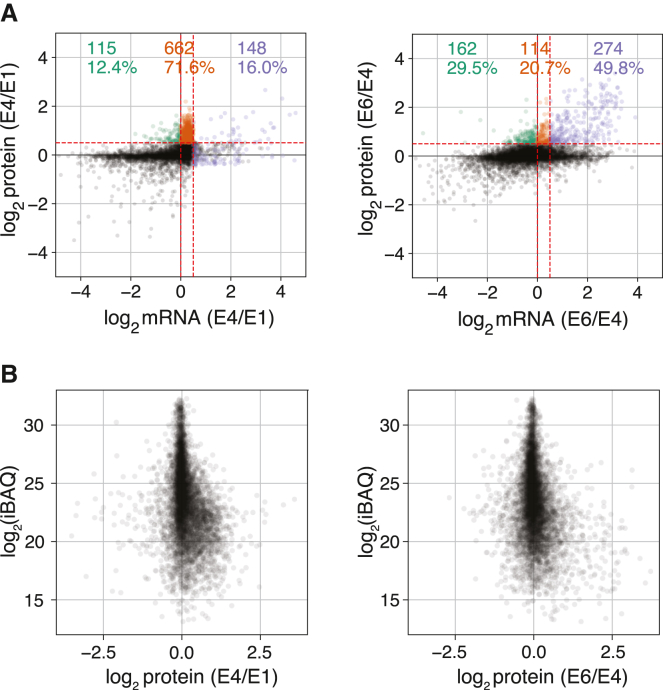
Relationship between protein and mRNA expression changes in embryos pre- and post-ZGA (A) Scatterplot comparing protein and mRNA expression changes in E1 to E4(left panel) and E4 to E6 (right panel). Proteins with increased expression levels (log2 fold change >0.5) are categorized based on corresponding mRNA expression changes: log2 fold change <0, between 0 and 0.5, and >0.5. The number and proportion of proteins in each category are shown from left to right. (B) Scatterplot of log2 protein fold changes (x axis) and log2 absolute protein quantities (y axis). The mean intensity-based absolute quantification value (iBAQ) across all embryo time points serves as a proxy for protein absolute quantities. The protein level changes from E1 to E4 (left) and E4 to E6 (right).

Earliest stages of embryogenesis are achieved with little to no amount of input from the newly transcribed gene product. Accordingly, previous studies have found that post-transcriptionally regulated increases in protein expression are essential for the progression of early embryogenesis.^23^ In *Drosophila*, it was found that both the pioneer factor, Zld, and the regulatory RBP, Smg, are upregulated based on the alleviation of the translation repression in pre-ZGA.^42^^,^^48^ Comparing the protein expression level change and the transcript expression level change, we found that during the earlier stage, E1 to E4, over 70% of genes with protein expression level increase (log2 fold change>0.5) were accompanied by only log2 mRNA fold change of 0–0.5, indicating that such increase is largely dependent on the post transcriptional regulation (Figure 4A). In the post-ZGA embryos, we find that only 20% of the genes increased in protein expression level without the change in transcript expression level. Surprisingly, we found that close to 30% of the genes with decreased transcript expression level increased in the protein expression level (Figure 4A). Previous study has recorded the genome-wide change in translational efficiency (TE) during the oocyte-to-embryo transition (OET).^49^ Our gene expression analysis measured time-course change of both transcript and protein expressions throughout the MZT for over 3000 genes whose TE changes were reported previously,^49^ although the analysis time points were not strictly aligned (Table S1). Among these genes, a majority (75%) of those with increased protein expression levels (log2 fold change >0.5) showed elevated TE (log2 fold change >0.5, from oocyte stage 14 to 0–1 h embryo). Intriguingly, less than 50% of the genes with increased protein expression levels demonstrated increased TE post-ZGA (from 0-1 h to 3–4 h embryos) (Figure S5A). Additionally, we observed that proteins with lower absolute levels tended to show relatively larger increase at the later time point, particularly pre-ZGA (Figure 4B). Combined with the observation that actively translated genes with increased protein levels have lower initial protein abundance, our analysis suggest that translational upregulation is influenced by baseline protein abundance (Figure S5B).

### Ubiquitome analysis reveals proteome under active degradation in *Drosophila* MZT

**Figure 5:**
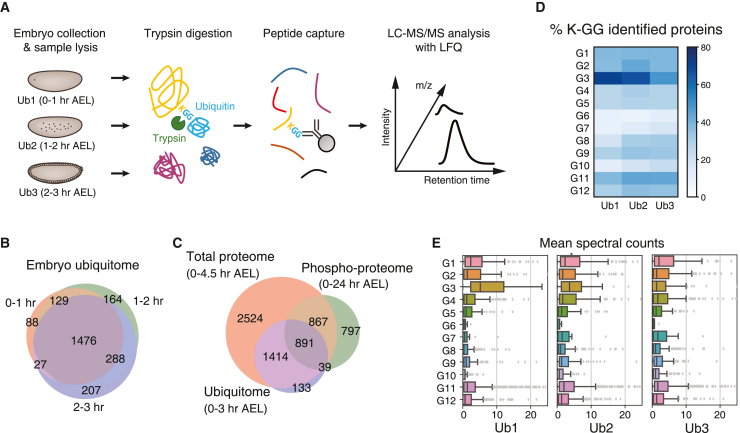
Ubiquitome analysis of the *Drosophila* embryos (A) Schematic representation of the ubiquitome analysis in *Drosophila* embryos collected at three distinct time points (*N* = 3). (B) Overlap between the ubiquitinated protein identification at three different time points. (C) Overlap between the protein identification in global, ubiquitome, and phospho-proteome analyses.^52^ (D) Heatmap displaying the fraction of proteins mapped to peptides with internal K-GG modifications across groups identified in the gene expression pattern analysis. (E) Mean spectral counts of ubiquitinated proteins for each cluster represented as boxplots. The boxplots show the median (center line), first and third quartiles (lower and upper box limits, respectively), and 1.5 times the interquartile range (whiskers).

We and others have found that distinct groups of protein expression levels are strongly downregulated at the beginning of embryogenesis based on maternally provided mechanisms.^50^^,^^51^ Previous studies also demonstrated that downregulation of distinct regulatory RBPs through ubiquitination-mediated protein degradation is essential for the progression of *Drosophila* MZT.^50^^,^^51^ Nonetheless, without the proteome-wide profiling of protein ubiquitination level, both the landscape of protein ubiquitination in *Drosophila* embryos and the impact on protein expression level changes remained largely unknown. We performed a proteome-wide analysis of the changes in protein ubiquitination in *Drosophila* embryo samples collected at three distinct time points to examine genome-wide relationship between the dynamics of protein ubiquitination level and respective change in protein expression level (Figure 5A). Our analysis identified ubiquitination sites within 2495 proteins. Both large overlap of ubiquitinated protein identifications and highly correlated intensities between each sample demonstrate the reproducibility of our ubiquitome analysis (Figures 5B and S6; Table S2). Notably, most of the ubiquitinated proteins intersected with our total proteome analysis, encompassing approximately 40% of the total embryo proteome suggesting that ubiquitination is widespread PTM in *Drosophila* MZT (Figure 5C). We also found that a comparable number of the proteins were reported to be phosphorylated in *Drosophila* embryos 0-24h AEL and there was approximately one-third of overlap^52^ (Figure 5C). Phosphorylation is known to be able to promote protein decay through the co-occurrence with protein ubiquitination.^53^^,^^54^^,^^55^ We identified potential targets for such phosphorylation mediated protein decay pathways in MZT. As aforementioned, the primary function of protein ubiquitination is the cue for protein decay through the recruitment to proteasome-mediated protein degradation pathway.^56^ We thus asked whether there are associations between the protein expression downregulation during MZT, observed in our total proteome analysis, and the dynamics of protein ubiquitination. The ubiquitome analysis identified over 70% of proteins belonging to G3 in 0–1 h samples. Despite the significant reduction in protein expression, ∼67 and ∼49% of the proteins were also identified in 1–2 h and 2–3 h embryos, respectively (Figure 5D). Moreover, peptide spectrum match counts of ubiquitinated peptides in G3 were greater than those in other groups, especially at 0–1 h (Figure 5E). Taken together, we found that the high level of protein ubiquitination was associated with maternally driven protein downregulation during *Drosophila* MZT.

**Figure 6:**
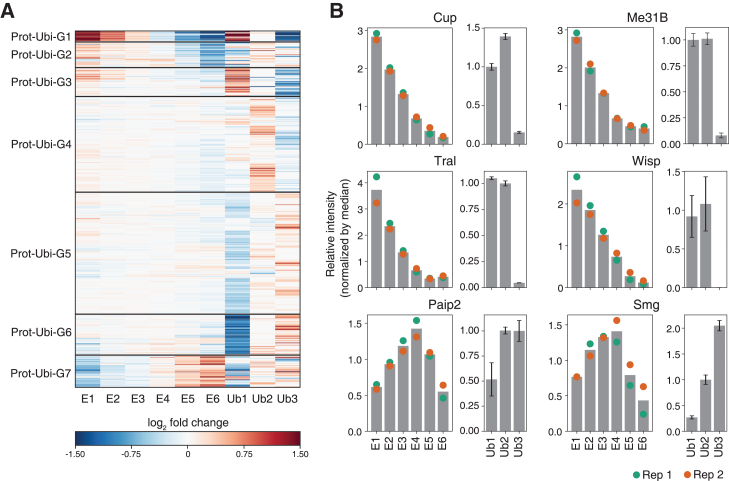
Dynamics of the protein ubiquitination and expression level changes (A) K-means clustering analysis of the total and ubiquitinated protein expression level changes. The color of the heatmap indicates the log2 fold change relative to the median of the total protein samples for “total” proteins, and relative to the median of the ubiquitinated protein samples for “ubiquitinated” proteins. Each gene was subjected to this log2 fold change calculation separately. (B) Total and ubiquitinated protein expression levels of selected genes. Left bar plots show the change in mean total protein expression at each time point. Green and orange dots indicate the total protein quantities in each replicate (*N* = 2). Right bar plots represent the mean expression level of ubiquitinated proteins (*N* = 3). Error bars indicate the standard error of the mean.

To further explore the quantitative changes in protein ubiquitination levels compared to protein expression levels, we employed k-means clustering. Our analysis revealed seven distinct groups based on the patterns of change in both total and ubiquitinated protein levels (Figure 6A). Prot-Ubi-G1 contained most of the aforementioned G3 proteins characterized by higher ubiquitination levels at 0–1 h with a strong decline in total protein expression. We observed dramatic change in both protein expression and protein ubiquitination levels for the key regulatory RBPs such as Cup, Me31B, Tral, and Wisp (Figure 6B). The result is also consistent with the previous reports,^50^^,^^51^ except for the Wisp whose ubiquitination was not reported previously (Figure 6B). Additionally, some proteins belonging to Prot-Ubi-G2 and G6 showed upregulated ubiquitination levels, whereas total protein expression levels were downregulated following ZGA (Figures 6B and S7). We also showed that the ubiquitination level of Smg was consistently upregulated, whereas there was a clear downregulation of total protein expression at the respective time points. This result is also consistent with the previous finding that the decay of Smg protein is mediated through the zygotic expression of Skp/Cullin/F-box-containing (SCF) complex proteins^51^ (Figure 6B). Intriguingly, we also found that the protein expression change of the Paip2 protein is highly similar to that of the Smg protein and there also is a consistent increase in ubiquitination level (Figure 6B). While recent studies reported the interaction between the Paip2 protein and the newly transcribed transcripts in *Drosophila* embryos, the role of Paip2 in *Drosophila* MZT is unknown.^57^^,^^58^ The protein is a homolog of the human protein with the same name and also interacts with the Pabp,^57^ suggesting that it may have a distinct role in the regulation of the mRNA translation in MZT which is also comparable to that of the Smg.^51^ We also found that many of well-known key regulatory RBPs in MZT, such as Ge-1, Brat, EIF4G, Capr, Pabp, Ago3, Not1, Vas, Bel, and Fmr1 are consistently downregulated while the levels of ubiquitination increased throughout the MZT (Figure S7). GO term analysis also revealed that various RNA-related terms, such as “P granule”, “cytoplasmic stress granule”, “mRNA 3′-UTR binding”, and “piRNA binding”, are enriched in Prot-Ubi-G1,2 and G6 (Figure S8). These genes are implicated in mRNA translation and stability control. Intriguingly, Vas, Fmr1, Cup, Me31B, and Tral, are also known to be components of oocyte-specific granules that are actively dissociated during MZT. The recent study also identified m6A-dependent formation of the Fmr1 specific granule that is also actively disassociated post-MZT in *Drosophila* embryos.^59^ In human cells, targeted protein ubiquitination is essential for the dissociation of stress granules and such regulatory mechanisms may also be conserved in *Drosophila*.^60^ Taken together, our results suggest that active change in protein ubiquitination level is responsible for the decay of key regulatory RBPs, many of which are fundamental to the post-transcriptional gene regulation during *Drosophila* MZT.

### Gene-specific post-transcriptional regulation is associated with RBPs

**Figure 7:**
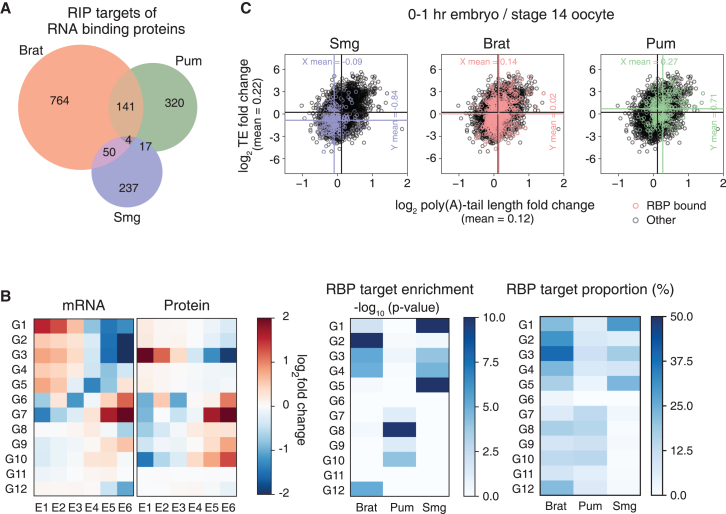
Characteristics of genes whose transcripts are bound by the regulatory RNA-binding proteins (RBPs), Brat, Pum, and Smg (A) Overlap of genes previously reported to be bound by Brat, Pum, and Smg proteins based on RIP analysis.^44^^,^^61^ (B) Heat maps showing RNA and protein expression levels (left, as shown in Figure 2A), *p*-values of Brat, Pum, and Smg target gene enrichment (middle), and proportions of Brat, Pum, and Smg target genes (right). The *p*-values for enrichment were calculated using the Fisher’s exact test. The proportions in the right panel indicate the number of RBP target genes divided by the total number of genes in each group. (C) Scatterplots showing the changes in poly(A)-tail length (x axis) and translation efficiency (TE) (y axis), reported previously in Eichhorn et al.^49^ Vertical and horizontal lines indicate the mean poly(A)-tail length and TE changes, respectively, of RBP target genes (colored) and other genes (black).

One of the key events in MZT is the clearance and translational repression of maternally provided transcripts. Regulatory mechanisms for such processes are both maternally provided, and thus active at the earlier part of MZT, and newly created through zygotic genome activation at the later part of MZT. In early embryos of *Drosophila*, several RBPs, including Brat,^61^ Pum,^61^ and Smg,^44^ are known to regulate the degradation and/or translation of target mRNAs based on the formation of distinct RNP complexes whose components are dynamically regulated at each stage of development.^26^^,^^27^^,^^42^ To further dissect their roles in MZT, we analyzed mRNA and protein expression patterns for mRNA targets of these RBPs, previously identified by RIP-seq/array in early embryos, (Figure 7B).^47^^,^^61^ This analysis revealed that Brat, Pum, and Smg had separate sets of targets sharing only a small number of genes (Figures 7A; Table S3). The targets of each RBP are enriched in distinct groups, classified by their expression profiles. Smg targets were strongly enriched in G1 and 5, which showed maternally programmed mRNA decay (Figure 7B). The Brat target genes are numerous and distributed in all the groups; however, enrichment is specific to G2-4, which also undergo maternally inherited mRNA decay (Figure 7B). Pum targets were prevalent in groups 8 and 10, with slightly increased mRNA levels in the initial stages of embryonic development before 2–3 h AEL, followed by a noticeable downregulation in expression levels (Figure 7B). At the individual gene level, Smg and Brat targets showed a decrease in mRNA abundance at approximately 1 h AEL, with Smg targets showing a more pronounced reduction than Brat targets (Figures S9A and S9B). However, only a small subset of Pum targets displayed mRNA downregulation at approximately 1 h AEL (Figure S9C).

Smg is also involved in translational repression prior to zygotic genome-derived transcription.^44^ Smg target genes have been reported to be less translated in 0–1 h AEL embryos^49^ (Figure 7C). Conversely, Brat targets did not exhibit translational downregulation (Figure 7C). In addition, the translational efficiencies of Pum targets increased, indicating that Pum binding did not lead to translational repression at the initial stage of embryogenesis (Figure 7C). We then analyzed whether Smg-mediated translational repression was correlated with the downregulation of protein levels by quantifying protein abundance in unfertilized eggs of wild-type and *smg* mutant. In the *smg* mutant, altered protein abundances were observed; however, a consistent trend of protein increase was not detected in the Smg target genes (Figure S9D; Table S4). The results suggest that the mutation of *smg* complex regulatory effects on the net protein expression level of the target genes.

### Transformation of RNP complexes in *Drosophila* MZT

**Figure 8:**
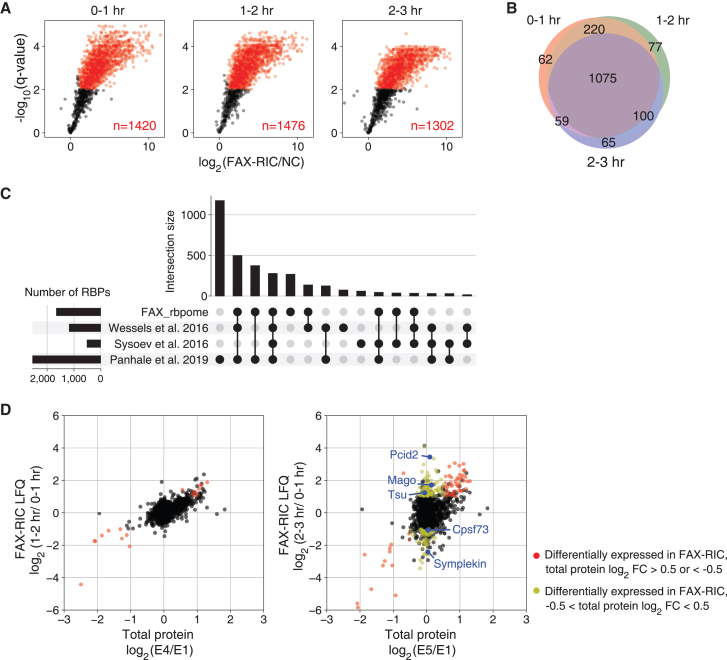
Formaldehyde crosslinking-based RNA-interacting proteome (FAX-RNA interactome) analysis in *Drosophila* embryos (A) High-confidence FAX-RNA interactome at different stages in *Drosophila* embryos (*N* = 3). Volcano plots displaying the log2 fold change of FAX over no crosslinking control (x axis) and –log10 *p*-value (y axis). Proteins with log2 fold change >1 and statistically significant enrichment over the no crosslinking control (BH-FDR <0.01) are highlighted in red. (B) Venn diagram showing the overlap of RNA interactomes defined in different stages of embryo samples. (C) Upset plot showing the number of proteins that are unique to or shared between FAX and previously reported *Drosophila* RNA interactomes.^62^^,^^64^^,^^65^ (D) FAX-RNA interactome and total protein expression level changes at the indicated time point in *Drosophila* embryo samples. Partially transparent red colored dots represent proteins with significant changes in FAX-RNA interactome enrichment (log2 fold change >1 and BH-FDR <0.05) that are attributable to corresponding changes in total protein expression levels (total protein log2 fold change >0.5 or < −0.5). Partially transparent yellow colored dots represent dynamic RNA-binding proteins with significant FAX-RNA interactome enrichment changes, despite minimal changes in total protein expression levels (−0.5 < total protein log2 fold change <0.5). Representative proteins are marked in blue, with their names labeled.

We highlighted the post-transcriptional regulation mediated by key regulatory RBPs. To elucidate the genome-wide changes in RNA-protein interactions without significant changes in total protein expression levels, we conducted a proteomic analysis of the mRNP complexes using formaldehyde crosslinking, which overcomes the potential limitations of the UV crosslinking method.^22^ UV transmittance into *Drosophila* egg cytoplasm is no more than 10% or at 5 μm from the egg surface, suggesting that UV crosslinking-based profiling of the RNA interactome would be heavily biased toward the mRNP complexes formed at the surface of the embryos.^62^^,^^63^ We collected RBPs at three time points in embryo samples (0–1 h, 1–2 h, and 2–3 h AEL) through formaldehyde treatment and performed a pull-down experiment using oligo-dT beads. In contrast to previous studies on RBP profiling in *Drosophila* and zebrafish MZT, our RNA interactome capture study was performed at three consecutive time points to delineate the RNP complex remodeling pre-ZGA and post-ZGA. The use of robust formaldehyde treatment conditions (4%, 10 min) enabled efficient crosslinking and complete embryo penetration. Thus, our experiments generated unbiased and quantitatively accurate profiles of changes in the RNA-binding proteome. LC-MS/MS analysis and quantitative comparison identified approximately 1400 proteins as RNA interactome at each time point (Figure 8A). The profiles of the RNA interactome largely overlapped between each time point and protein intensities were highly reproducible indicating that our RNPome profiling was both comprehensive and reliable (Figures 8B and S10). Approximately 80% of our RNP profiles were consistent with RBPs reported in previous RNA interactome capture experiments performed in *Drosophila* embryos and S2 cells^62^^,^^64^^,^^65^ (Figure 8C).

Finally, we performed statistical analysis to determine the proteins that exhibited significant changes in RNA association at different MZT stages. We found 37 and 370 proteins with statistically significant changes in capture efficiency (BH-FDR <0.05 and log2 fold change >1) at 1–2 h and 2–3 h, compared to that at 0–1 h (Figure 8D). Most of the 37 differentially enriched proteins in 1–2 h embryos were aligned with either upregulation or downregulation of the protein expression level from 0–0.5 h to 1.5–2 h embryos (Figure 8D). These results demonstrated that significant changes in protein expression were immediately reflected in their RNA regulatory capacities. However, a greater number of proteins were identified with significant changes in RNA interactome capture efficiencies in 2–3 h embryos, and more than two-thirds of such proteins had a log2 fold change of less than 0.5 in the total protein expression, indicative of altered association with RNA (Figure 8D). Among the RBPs with the most significant upregulation in RNA association was Pcid2, which plays a role in the processing of newly transcribed RNA.^66^ We also observed increased levels of EJC complex proteins, such as Mago and Tsu, during RNA interactome capture^67^ (Figure 8D). In contrast, our analysis found that most other nascent transcript-associated RBPs had either unchanged or even reduced RNA-binding activity post-ZGA (Table S5). Thus, the results suggest that there may be yet unknown distinct regulatory mechanisms or features that mediate the binding of Pcid2 and EJC complex proteins to newly synthesized RNAs from ZGA. Interestingly, among the downregulated RBPs in the 2–3 h embryos were Sym and Cpsf73, which are involved in the processing of newly transcribed RNAs^68^ (Figure 8D). The CPEB-mediated cytoplasmic polyadenylation complex containing Sympk and Cpsf73, the *X. laevis* orthologs of Sym and Cpsf73, respectively, is known to regulate the poly-A tail length in *X. laevis* oocytes and embryos.^69^ We recently reported that RNP complexes containing Sympk and Cpsf3 are dynamically regulated during embryonic development in *X. laevis*.^22^ Our RNA interactome analysis of early *Drosophila* embryos suggested that developmental stage-specific transformations of Sym- and Cpsf-containing RNP complexes are evolutionarily conserved.

## Discussion

The initial step of early embryonic development involves various biological processes that are tightly regulated within a short period. A high-quality catalog of gene expression is essential for a comprehensive understanding of the regulatory mechanisms of early development. Genome-wide analysis of gene expression has been mostly performed at the mRNA level for over a decade; however, more recently, a proteomic analysis-based draft of protein expression changes in several different model organisms has been reported.^32^^,^^35^^,^^42^ Prior to our study, most representative proteogenomic study performed in Drosophila was done for the whole span of embryogenesis with broader time windows (1 h time points) and the protein expression levels were measured based on the label free quantification method. In this study, we utilized protein tagging and prefractionation to achieve both reliable quantification and in-depth protein profiling. We also focused on the stage of MZT. As a result, we provide reliable and extensive transcriptomic and proteomic data from the earliest stages of *Drosophila* embryogenesis, systematically addressing the changes in the gene expression levels. The consecutive time points with 30-min intervals in our analysis allowed us to precisely track gene expression levels, providing insight into the dynamic orchestration of regulatory mechanisms during the MZT. We also found that our results were highly comparable to those of a separate study single-embryo transcriptome analysis. These findings suggest that our results could serve as a standard for global protein expression level changes during the earliest stages of *Drosophila* development in future studies.

In our study, mRNA and protein abundance data were generated in parallel from the same samples, enabling proper analysis of their correlation. Interestingly, the decreasing pattern of mRNA expression only partially matched the decrease in protein abundance during early embryonic development. This discrepancy suggests that the degradation of maternal mRNA does not cause a decrease in protein abundance during early development. In mammalian cells, it was reported that the synthesis rate and half-life of mRNA are much lower than those of protein.^10^^,^^11^^,^^70^ The massive decay of maternally deposited mRNAs may be critical because mutations in several key factors that mediate maternal mRNA decay, such as Smg, Brat, and Pum, lead to severe developmental arrest.^71^^,^^72^^,^^73^ Maternal RNA degradation is conserved in other animal models, although the common molecular mechanisms are unknown. In zebrafish, uridylation of maternal mRNA promotes mRNA degradation, and the depletion of terminal uridylyl transferase leads to a delay in maternal mRNA clearance and developmental defects.^29^ In another study, a large proportion of zebrafish maternal mRNAs were reported to be m6A-methylated, whose degradation is mediated by m6A-binding protein Ythdf2. The loss of Ythdf2 function impedes zygotic genome activation, resulting in developmental delays. These findings demonstrate that maternal mRNA clearance is essential for normal embryonic development. As we have demonstrated, mRNA decay during MZT alone does not result in decrease in the corresponding protein expression level. On the other hand, further analysis found that, in pre-ZGA embryos, increase in protein expression level was largely exclusive to the genes whose transcript expression level change from E1-E4 was log2 fold change of 0–0.5. The results suggest that while transcript decay does not result in significant downregulation of protein expression level, removal of maternally stockpiled transcripts can be an underlying mechanism to the preclusion of the protein expression level upregulation for the respective genes. Both transcriptome and proteome analysis techniques have evolved to categorize gene expression levels within a single cell and subcellular organelle and such techniques were also applied to the context of *Drosophila* embryogenesis.^29^^,^^74^^,^^75^^,^^76^^,^^77^ Not only the further analysis of our result based on the integration of such data but also future study based on the application of such methods will advance our understanding of *Drosophila* embryogenesis.

Among the genes with decreased mRNA expression, a small subset showed a dramatic decline in protein abundance. Protein downregulation appeared for the progression of the earliest two stages that we analyzed, suggesting that protein decay mechanisms are activated immediately after egg-laying. Further analysis also revealed that many of the respective genes have important functions in oocyte maturation and egg activation. The rapid and substantial decrease in proteins for the selected genes implied the activation of targeted protein degradation. Several oocyte-derived proteins are downregulated through the ubiquitin-proteasome pathway in *C. elegans*, *Drosophila*, *Xenopus*, and mice,^78^^,^^79^^,^^80^ which is essential for early embryonic development. In *Drosophila*, Me31B, Tral, and Cup, the RBPs of a maternal RNP complex, which belongs to group 3, are cleared by CTLH E3 ubiquitin ligase.^51^ In this study, we performed ubiquitome analysis in *Drosophila* MZT and found that most group 3 proteins were highly ubiquitinated, corresponding to their protein decay. We also revealed that many regulatory RBPs are highly ubiquitinated, and their exact roles and functions require further investigation.

As mentioned above, RBPs are critical factors that determine the landscape of gene expression dynamics in *the Drosophila* MZT. While previous studies have also revealed the RNA interactome profile in *Drosophila* embryos, they were conducted based on UV crosslinking, likely resulting in the underestimation of total RBPs and a significant bias toward the RNPs formed at the surface of embryos. Here, we report a highly comprehensive profile of proteins associated with mRNA transcription at three different stages of the MZT. The distinct changes in RBPs warrant further research into the underlying mechanisms that regulate their stage-specific RNA associations throughout early *Drosophila* embryogenesis. We and others have developed the proteomics based method that can profile RNA-protein interaction sites with the resolution of either tryptic peptide resolution or single amino acid residue.^22^^,^^81^^,^^82^^,^^83^^,^^84^ Notably, peptide level FAX-RIC method can be readily applied to the Drosophila embryos. Future study based on such method will reveal the stage and context specific changes in the RNA binding domains throughout the progression of *Drosophila* MZT.

### Limitations of the study

Our study conducted transcriptome and proteome analyses on pooled embryos, which were collected over a specific time period and incubated to reach the desired time points. Therefore, the study lacks the time resolution and precision that can be achieved through single-embryo analysis.^40^ This approach, however, enabled us to perform transcriptome and proteome analyses simultaneously on the same samples, facilitating the direct comparison of RNA and protein dynamics in *Drosophila* MZT. Multiple sample collection events and the large number of embryos collected during each preparation inherently normalized the data, ensuring relatively accurate measurements of stage-specific gene expression dynamics. However, it is important to note that the accuracy of later time-point samples may be relatively compromised, as slight shifts in initial sample collection times are inevitably amplified during later stages. In addition, the broader time intervals at later time points increased variability compared to the short intervals at earlier stages.

Gene expression control during development can exhibit cell type specificity and may also be highly localized within a cell, particularly during the earlier stages of development.^25^^,^^85^ Our study primarily focused on developmental stages prior to cellularization, therefore the recorded changes are likely representative of a single dominant pattern of gene expression level changes —or lack thereof— that occur throughout the embryos. However, these changes may also be an averaged representation of multiple distinct gene expression dynamics in different regions of the embryos. To achieve a more refined understanding of gene expression control in *Drosophila* embryos, future studies incorporating both single-cell and *in situ* proteome analyses will be essential.^86^

## Resource availability

### Lead contact

Further information and requests for resources and reagents should be directed to and will be fulfilled by the lead contact, Dr. Jong-Seo Kim (jongseokim@snu.ac.kr).

### Materials availability

This study does not generate unique reagents.

### Data and code availability

• All the original LC-MS/MS datasets and related identification files used to support this paper have been deposited to the ProteomeXchange Consortium (http://proteomecentral.proteomexchange.org) via the PRIDE partner repository with the dataset identifier, PRIDE: PXD047784, PXD057383. The original FASTQ files from the RNA-seq experiments in this study are available from the NCBI Gene Expression Omnibus (GEO) database under accession number GEO: GSE255926.
• All computer code used in this study is available at https://doi.org/10.5281/zenodo.15857949.

## Acknowledgments

We are grateful to Dr. Chunghun Lim for providing the anti-Me31B and anti-PABP antibodies. We also thank the laboratory members for their insightful discussions and assistance. This work was supported by the 10.13039/501100003725National Research Foundation of Korea (NRF-2019M3E5D3073092 and NRF-2021R1A2C4002421 to M.L., NRF-2022M3A9I2082294 and RS-2024-00343424 to J.-S.K., NRF-2022R1C1C2003822 to Y.N.); Institute for Basic Science from the Ministry of Science and ICT of Korea (IBS-R008-D1 to J.-S.K.); Faculty Supporting Fund from 10.13039/100004358Samsung Electronics Co., Ltd. (IO220819-02121-01 to J.-S.K.); and 10.13039/100015506POSCO TJ Park Foundation (POSCO Science Fellowship to M.L.).

## Author contributions

Conceptualization, M.L. and J.-S.K.; methodology, Y.N., Y.C., M.L., and J.-S.K.; formal analysis, Y.N. and Y.C.; investigation, Y.N., Y.C., T.T.M.N., H-A.P.-B., J.K., M.L., and J-S.K.; writing – original draft: Y.N., Y.C., M.L., and J.-S.K.; supervision, V.N.K., M.L., and J.-S.K.; funding acquisition, Y.N., M.L., and J.-S.K.

## Declaration of interests

The authors declare no competing interests.

## STAR★Methods

### Key resources table

**Table:** 

REAGENT or RESOURCE	SOURCE	IDENTIFIER
**Antibodies**		

anti-Ago1	Abcam	ab5070; RRID:AB_2277644
anti-HSP83 (anti-HSP90 antibody)	Cell Signaling Technology	#4874; RRID:AB_2121214
anti-RpS6	Cell Signaling Technology	#2317; RRID:AB_2238583
anti-Wisp	Lee et al.^24^	N/A
anti-Me31B	Lee et al.^87^	N/A
anti-PABP	Lee et al.^87^	N/A

**Chemicals, peptides, and recombinant proteins**		

Protease inhibitor cocktail	Calbiochem	539134
RNasin RNase inhibitor	Promega	N2111
TRIzol	Thermo Fisher Scientific	#15596018
Trypsin	Promega	#20233
Tandem mass tagging (TMT) 10-plex reagent	Thermo Fisher Scientific	#90110

**Critical commercial assays**		

SuperSignal West Pico chemiluminescent substrate	Thermo Fisher Scientific	
PTMScan® HS Ubiquitin/SUMO Remnant Motif (K-ε-GG) Kit	Cell Signaling Technology	#59322

**Deposited data**		

Raw data of RNA-sequencing	This paper	GEO:GSE255926
Raw data of tandem mass tag mass spectrometry	This paper	ProteomeXchange:PXD047784

**Experimental models: Organisms/strains**		

Drosophila melanogaster: *w*^*1118*^	Bloomington Drosophila Stock Center	BDSC; 3605
Drosophila melanogaster: *smg**^1^*	Bloomington Drosophila Stock Center	BDSC; 5930
Drosophila melanogaster: *Df(Scf)*	Bloomington Drosophila Stock Center	BDSC; 4500
Drosophila melanogaster: *tud**^1^*	Kyoto Drosophila Stock Center	DGRC; 106505

**Software and algorithms**		

In-house scripts for RNA-seq / tandem mass tag mass spectrometry data	This paper	doi:10.5281/zenodo.15857949
FASTX-Toolkit (version 0.0.13)	Hannon lab at Cold Spring Harbor Laboratory	http://hannonlab.cshl.edu/fastx_toolkit/
RSEM (v1.3.1)	Li et al.^88^	https://github.com/deweylab/RSEM
STAR (v2.5.3a)	Dobin et al.^89^	https://github.com/alexdobin/STAR
ProteomeDiscoverer (version 2.4)	Thermo Fisher Scientific	https://www.thermofisher.com/us/en/home/industrial/mass-spectrometry/liquid-chromatography-mass-spectrometry-lc-ms/lc-ms-software/multi-omics-data-analysis/proteome-discoverer-software.html
MaxQuant (v1.6.15.0)	Tyanova et al.^90^	https://www.maxquant.org/
Perseus software (v2.0.11)	Tyranova et al.^91^	https://maxquant.net/archive/perseus
Python package scipy (version 1.5.2)	SciPy Steering Council	https://scipy.org/
TopGO package (v2.46.0)	Package ‘topGO’	https://doi.org/10.1093/bioinformatics/btl140

**Other**		

Ziptip C18 P10	Millipore	ZTC18S
Oligo d(T)25 Magnetic Beads	New England Biolabs	S1419S

### Experimental model and study participant details

#### *Drosophila* genetics and embryo collection

The fly lines *w*^*1118*^, *smg,*1 and *Df(Scf)* were obtained from the Bloomington *Drosophila* Stock Center (Bloomington, IN, USA), whereas tud^1^ was obtained from the Kyoto Stock Center (Kyoto, Japan). *w*^*1118*^ was used as the wild-type control. *smg*^*1*^*/Df(Scf)* has been previously described as a null allele of *smg*.^42^ Unfertilized activated eggs were produced from *w*^*1118*^ virgin females mated with sterile males (sons of *tud*^*1*^ mothers).^92^ Fly eggs and embryos were collected from 100 to 150 flies aged 4–7 days post-hatching, in a 7.5 cm (diameter) × 10 cm (height) cage. The collections were performed on grape juice plates for the designated time frame at 25 °C. The first collection at 0.5 h or 1 h each day was discarded to exclude embryos that had been retained within the female oviduct for a prolonged time. In preparing embryo samples, we analyzed the hatching rate for each batch. We confirmed that overall embryonic development proceeded as expected, culminating in hatching at 22–24 h after egg laying (AEL).

#### *Drosophila* sample preparation

To prepare RNA and protein samples, fly embryos and unfertilized eggs were dechorionated with bleach and frozen in liquid nitrogen for the further processing. We added the lysis buffer to the frozen embryos, adjusting the amount based on the approximate volume of embryos collected (50 μL of lysis buffer per 20 μL of embryos in a tube). The embryos were then homogenized in lysis buffer [8 M urea, 50 mM ammonium bicarbonate, 1× protease inhibitor cocktail (Calbiochem, San Diego, CA, USA), and RNasin RNase inhibitor (Promega, Madison, WI, USA)] on ice. After the lysate of several (up to eight) batches was combined for each replicate (*N* = 2), 10% was used for RNA extraction, while the remaining 90% was used for proteome analysis. To isolate the total RNA, TRIzol reagent was immediately added to an aliquot of the fresh lysate. The remaining lysates were frozen for mass spectrometric (MS) analysis.

### Method details

#### RNA sequencing (RNA-seq) analysis

Total RNA isolated from fly embryos or unfertilized eggs using TRIzol was analyzed with an Agilent 2100 Bioanalyzer for quality checks. RNA-seq libraries were generated by Macrogen, Inc. (Seoul, South Korea) using the TruSeq Stranded RNA LT kit.

#### Western blotting

Fly embryo lysates were prepared via homogenization in the lysis buffer (8 M urea, 50 mM ammonium bicarbonate, and 1× protease inhibitor cocktail). The supernatant was obtained via centrifugation at 13,500 *g* for 5 min at 4 °C. Proteins were separated via SDS-PAGE and transferred to Whatman Protran nitrocellulose membranes (GE Healthcare, Chicago, IL, USA). Membranes were blocked in 5% skim milk/1× PBST (phosphate-buffered saline, 0.1% Tween 20) for 1 h at room temperature and incubated with primary antibodies overnight at 4 °C. After several washes, membranes were incubated with horseradish peroxidase-conjugated secondary antibodies. Protein bands were detected using the Super Signal West Pico chemiluminescent substrate (Thermo Fisher Scientific Inc., Waltham, MA, USA) on a ChemiDoc Imaging System. Primary antibodies used for western blotting were: Anti-Ago1 (ab5070; Abcam, Cambridge, UK), anti-HSP83 (anti-HSP90 antibody, #4874; Cell Signaling Technology, Danvers, MA, USA), anti-Wisp,^24^ anti-RpS6 (#2317; Cell Signaling Technology), anti-Me31B,^87^ and anti-PABP.^87^

#### MS sample preparation for global and ubiquitome analysis

Embryo samples were lysed in lysis buffer (8 M urea, 50 mM ammonium bicarbonate, 1× protease inhibitor cocktail (Calbiochem, San Diego, CA, USA), and RNasin RNase inhibitor (Promega, Madison, WI, USA) and first treated with 10 mM DTT (Sigma-Aldrich, St. Louis, MO, USA). Free thiol groups were alkylated with 40 mM Iodoacetamide (Sigma-Aldrich). Samples were diluted with 50 mM ammonium bicarbonate buffer to a final urea concentration of 1 M. The proteins were digested overnight at 37 °C using sequencing-grade modified trypsin (Promega) at a 1:50 enzyme-to-protein ratio (w/w). For global protein profiling analysis, equal amounts of peptides for each time point were labeled with tandem mass tagging (TMT) 10-plex reagent (Thermo Fisher Scientific), fractionated, and concatenated into 24 fractions, as previously described.^93^ For ubiquitome analysis, the desalted peptides were subjected to immunoprecipitation. Ubiquitinated peptide enrichment was performed according to the protocol provided by the manufacturer of PTMScan HS Ubiquitin/SUMO Remnant Motif (K-ε-GG) Kit (Cell Signaling Technology). The resulting peptides were purified using a Ziptip C18 P10 (Millipore, Burlington, MA, USA) and subjected to MS analysis.

#### Liquid chromatography coupled mass spectrometry (LC-MS/MS) analysis

The column and instrumentation settings for the liquid chromatography analysis were identical to those described in a previous study.^93^ MS analysis of global TMT was performed using an Orbitrap Fusion Lumos mass spectrometer (Thermo Fisher Scientific). The flow rate of the UPLC system was 300 nL/min for all the analyses. For global TMT profiling, a linear gradient of solvent A [H_2_O with 0.1% formic acid (Merck)] and solvent B (100% acetonitrile with 0.1% formic acid) was used; solvent B was increased from 5 to 10% for the initial 10 min, 10 to 40% for the next 230 min, and 40 to 80% for the next 10 min. The total run time of the SPS-MS3 analysis was 240 min with the following setup for MS acquisition: MS1 with 120 K resolution, 5e5 AGC target value, 30% RF, and maximum injection time of 100 ms; MS2 with 15 K resolution, 1e5 AGC target value, HCD collision energy of 30%, and maximum injection time of 60 ms; MS3 with 50 K Orbitrap resolution, 5e4 AGC target value, HCD collision energy of 55%, and maximum injection time of 150 ms.

MS analysis of the ubiquitome and FAX RNA-associated proteome profiling was performed using an Orbitrap Eclipse mass spectrometer. For the ubiquitome analysis, a linear gradient of solvent A (H_2_O with 0.1% formic acid) and solvent B (100% acetonitrile with 0.1% formic acid) was used; solvent B was increased from 5 to 10% for the initial 10 min, 10 to 40% for the next 100 min, and 40 to 80% for the next 10 min. The total runtime of MS2 was 120 min, and the following setup was used for MS acquisition: MS1 was acquired with 120 K Orbitrap resolution, 4e5 AGC target value, 30% RF, and maximum injection time was set to auto; MS2 was acquired with 15 K Orbitrap resolution, 5e4 AGC target value, HCD collision energy of 30%, and maximum injection time set to auto.

For FAX RNA-associated proteome analysis, a linear gradient of solvent A (H_2_O with 0.1% formic acid) and solvent B (100% acetonitrile with 0.1% formic acid) was used; solvent B was raised from 5 to 10% for the initial 10 min and 10 to 40% for the next 60 min. The total runtime of MS2 was 80 min, and the following setup was used for MS acquisition: MS1 was acquired with 120 K Orbitrap resolution, RF 30%, 4e5 AGC target value, and maximum injection time set to auto; MS2 was acquired with 15 K Orbitrap resolution, 5e4 AGC target value, HCD collision energy of 30%, and maximum injection time set to auto.

#### FAX RNA-associated proteome profiling

Fly embryos were collected and dechorionated using bleach. After washing several times with distilled water, the embryos were fixed in 4% formaldehyde/1× PBS with Heptane (1:1) for 10 min, subjected to devitellinization in MeOH-fixed embryos, washed with 1× PBS, and frozen in liquid nitrogen. Fixed embryos were homogenized in a lysis buffer followed by oligo-dT bead pull-down. Lysis buffer was composed of 0.5% (w/v) lithium dodecyl sulfate (LDS), 500 mM lithium chloride (LiCl), 10 mM Tris (pH 7.5), 2 mM EDTA, and 5 mM dithiothreitol (DTT) (all Sigma). Oligo-dT bead (NE Biolabs) was added to the lysate and incubation were done with over and over rotation for 1 h at room temperature. Beads were separated from the embryo lysate using DynaMag (Thermo scientific) and washed twice in each buffer, the lysis buffer, low LDS lysis buffer (0.1% (w/v) LDS, 500 mM LiCl, 10 mM Tris (pH 7.5), 2 mM EDTA, and 5 mM DTT, high salt buffer (500 mM LiCl, 10 mM Tris (pH 7.5), 2 mM EDTA, and 5 mM DTT) and low salt buffer (200 mM LiCl, 10 mM Tris (pH 7.5), 2 mM EDTA, and 5 mM DTT). Oligo-dT beads were then incubated with Turbo DNase (Thermo Scientific) in Turbo DNase buffer supplemented with 200 mM of LiCl, for 30 min with over and over rotation at RT. Beads were then washed twice with each wash buffer. Elution of the poly(A) RNA by heat was done twice in TE buffer, by incubation on Thermomixer C (Eppendorf) at 65°C for 3 min with mixing at 800 rpm. Protein sample preparation for MS analysis was performed following the protocol described for HeLa cell RNA interactome capture.^22^

### Quantification and statistical analysis

#### Data analysis of RNA-seq

Resulting sequence reads underwent pre-processing using the FASTX-Toolkit (http://hannonlab.cshl.edu/fastx_toolkit/, version 0.0.13), including 3′ adaptor sequence removal, followed by filtering out of the sequence reads with artifacts (e.g., long homopolymer) or those of low quality (Phred quality <30 in >5% of nucleotides). In addition, sequence reads shorter than 18 nt were removed after 3′ adaptor removal. To obtain the read count for each gene, we prepared indexed genome files for RSEM^88^ (v1.3.1, https://doi.org/10.1186/1471-2105-12-323) and STAR^89^ (v2.5.3a, 10.1093/bioinformatics/bts635), with the UCSC Genome Browser dm6 genome sequence and RefGene annotation. Individual gene read counts and normalized counts (TPM) were obtained using the ‘rsem-calculate-expression’ command with ‘--star’ and ‘--forward-prob 0.0’ parameters.

#### Clustering and GO term enrichment analysis

To perform clustering analysis of the RNA and protein quantities of the embryo samples, the following procedures were performed: To prevent division by zero during fold-change calculations, a pseudo-value of 1 was added to all RNA-seq TPM values and LC-MS3 TMT quantities. Subsequently, the RNA and protein quantities were log2-transformed and adjusted by subtracting the median RNA or protein quantities for each gene. K-means clustering of embryonic mRNA and protein expressions was conducted with spatial and cluster modules of the python package scipy (version 1.5.2), based on median-normalized RNA and protein quantities. The 5,709 genes detected both at the mRNA and protein levels were subjected to the clustering analysis, with the following expression cutoffs: For RNA-seq, a gene was considered ‘expressed’ if it had more than 500 reads at any time point or an average of more than 100 reads in either of the two replicates; for proteins, a false discovery rate (FDR) of less than 1% at the protein level and the identification of two or more unique peptides mapped to the protein group were required. We performed the same procedure for the clustering analysis of total embryos and LFQ-measured embryo ubiquitomes, except that LFQ-measured embryo ubiquitomes were used instead of RNA quantities.

For GO term enrichment analysis, the TopGO package^94^ (v2.46.0, https://doi.org/10.1093/bioinformatics/btl140) was used, with GO annotations curated by org.Dm.e.g.,.db (v 3.14.0). To mitigate bias resulting from the GO terms of highly expressed genes in all embryos and capture the true gene set differences between clusters, we applied the 5,709 genes used for the clustering analysis as the statistical background of this analysis. The resulting enrichment *p*-value for each GO term was corrected using the weight01 algorithm, which helps reduce the overrepresentation of redundant GO terms.

#### Protein identification and quantification of LC-MS3 data

*Drosophila* melanogaster reference protein sequences were obtained from the NCBI RefSeq invertebrate proteome (downloaded on May 9^th^, 2018). To identify and quantify proteins from LC-MS3 data, RAW files were processed with Proteome Discoverer (version 2.4) with isotope impurity correction for TMT labels (Thermo Fisher Scientific TMT10plex Isobaric Label Reagents, lot number PB1991188D for replicate 1, PF201859 for replicate 2), by setting “Apply Quan Value Corrections” = True in the “reporter ions quantifier” node. Only peptides that uniquely matched a single protein group were used for protein quantification. We utilized Sequest HT and Percolator with the default setting.

#### Protein identification and quantification of LC-MS/MS data

The same reference proteome used for LC-MS3 data was used for the analysis of FAX-RIC LFQ and Ubiquitome LFQ data. MaxQuant^90^ (v1.6.15.0, https://www.maxquant.org/) was used for label-free quantification, based on the LFQ algorithm. MaxQuant search and quantification were performed using standard settings. Only the proteins with the peptide quantified at two out of three replicates in a time point was considered for quantitative Ubiquitome analysis. Average of three or two replicates were obtained and summed and considered as protein ubiquitination level. Peptide identified with terminal lysine modified with diGly was considered as an artifact and removed from quantification analysis. For the determination of Formaldehyde crosslinking based RNA interactome profile, the proteins with LFQ value obtained from all Formaldehyde crosslinked proteome analysis were used for further analysis based on the use of statistical test provided in Perseus software^91^ (v2.0.11, https://maxquant.net/archive/perseus). Missing LFQ values within the control sample analysis were imputed with a normal distribution shifted by −4 and sharpened with a standard deviation factor of 0.3. Student’s t-test was performed to test if any log2 fold-change ratio is different from 0 and protein groups with Benjamini Hochberg FDR <0.01 were considered as RNA interactome. For differentially enriched RNA binding protein determination, Benjamini Hochberg FDR <0.05, was used.

## Supplemental information

Document S1. Figure S1–S10

Data S1. Original images for blots, related to Figure2D

Table S1. Total mRNA and protein expression level change in embryos and eggs of Drosophila, related to Figure 2

Table S2. Ubiquitome change in Drosophila MZT, related to Figure 5

Table S3. Genes targeted by the regulatory RBPs and changes in gene expression level, related to Figure 7

Table S4. Proteome change upon the depletion of Smg protein, related to Figure 7

Table S5. FAX-RIC in Drosophila MZT
