# Supplementary material for: Gene expression dynamics before and after zygotic gene activation in Drosophila early embryogenesis
Source: iScience. 2025 Aug 7;28(9):113272. doi: 10.1016/j.isci.2025.113272 (PMC12396305; doi:10.1016/j.isci.2025.113272)
Supplement: Document S1. Figure S1–S10 [file mmc1.pdf]

iScience, Volume *28*

Supplemental information

Gene expression dynamics before and after zygotic gene activation in *Drosophila* early embryogenesis

Yongwoo Na, Yeon Choi, Thi Thanh My Nguyen, Hoang-Anh Pham-Bui, Jeesoo Kim, V. Narry Kim, Mihye Lee, and Jong-Seo Kim

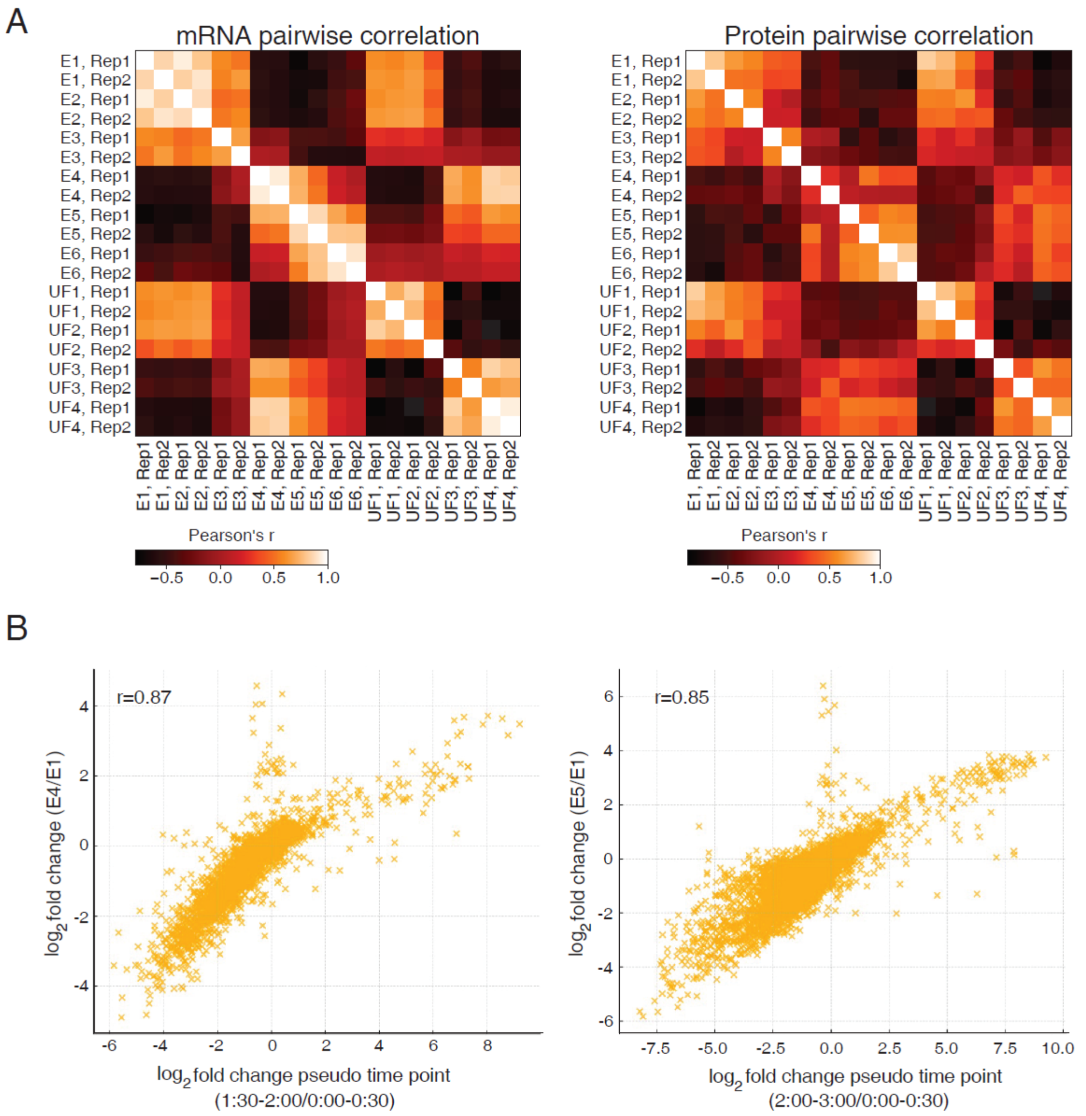

Figure S1. Reproducibility of gene expression dynamics measurement in *Drosophila* MZT, Related to Figure1

(A) The reproducibility of mRNA and protein expression was assessed using Pearson's correlation coefficients. E1–E6 embryo samples and UF1–UF4 unfertilized egg samples are illustrated in Figure 1A. (B) Comparison of the transcript expression level changes from our data and single-embryo transcriptome data by Pérez-Mojica et al. (2024). The $\log_2$ fold changes from 0:00-0:30 AEL to 1:30-2:00 AEL (left panel) and from 0:00-0:30 AEL to 2:00-3:00 AEL (right panel) are shown. From the single-embryo sequencing analysis, which aligned data to 84 different time points spanning 0-3hr period, we selected the corresponding data points and calculated the median value to estimate transcript expression level changes[45].

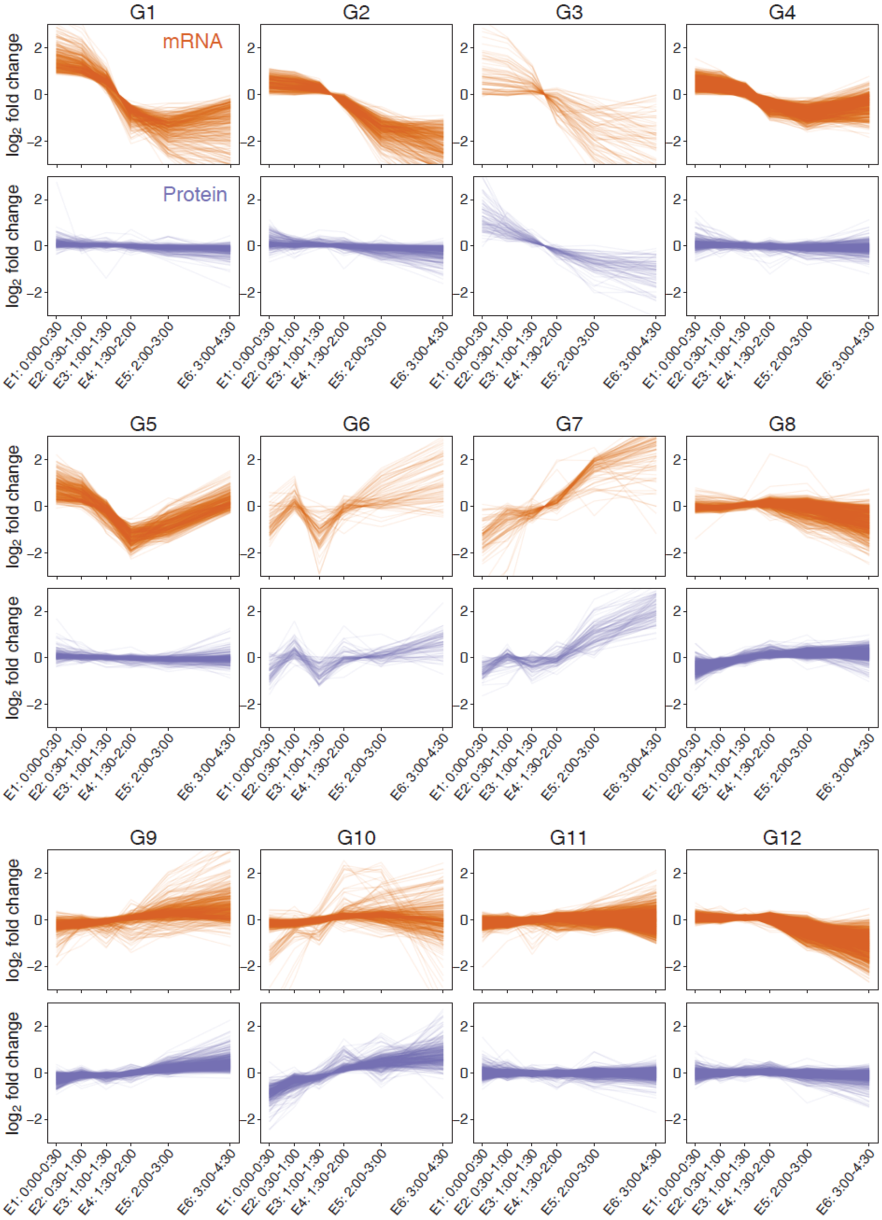

Figure S2. The pattern of gene expression dynamics in each group, Related to Figure 2

Log2-transformed mRNA and protein expression dynamics, normalized by the median mRNA and protein expression levels of each gene.

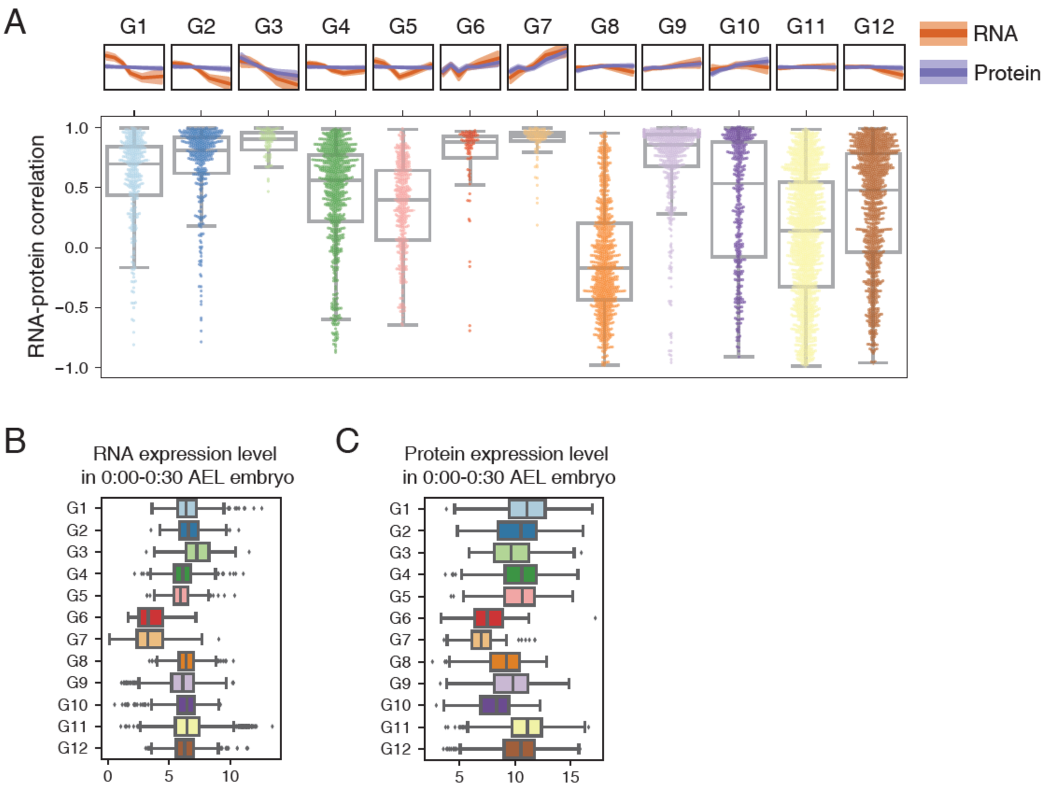

Figure S3. mRNA and protein expression dynamics versus each other, and absolute quantity in 0:00-0:30 AEL embryos, Related to Figure 2

(A) Box and beeswarm plots of RNA-protein correlation coefficients for individual gene, in each group. Small line plots above indicate the median (dark line) and standard deviation (light line) of RNA and protein temporal dynamics. Box plots show the median (center line), first and third quartiles (lower and upper box limits, respectively), and 1.5 times the interquartile range (whiskers). (B) Box plots showing the mean absolute quantities of mRNA (left) and protein (right) at E1 (0:00-0:30 embryo) for each group. Box plot elements (median, quartiles, and whiskers) are represented as in (A).

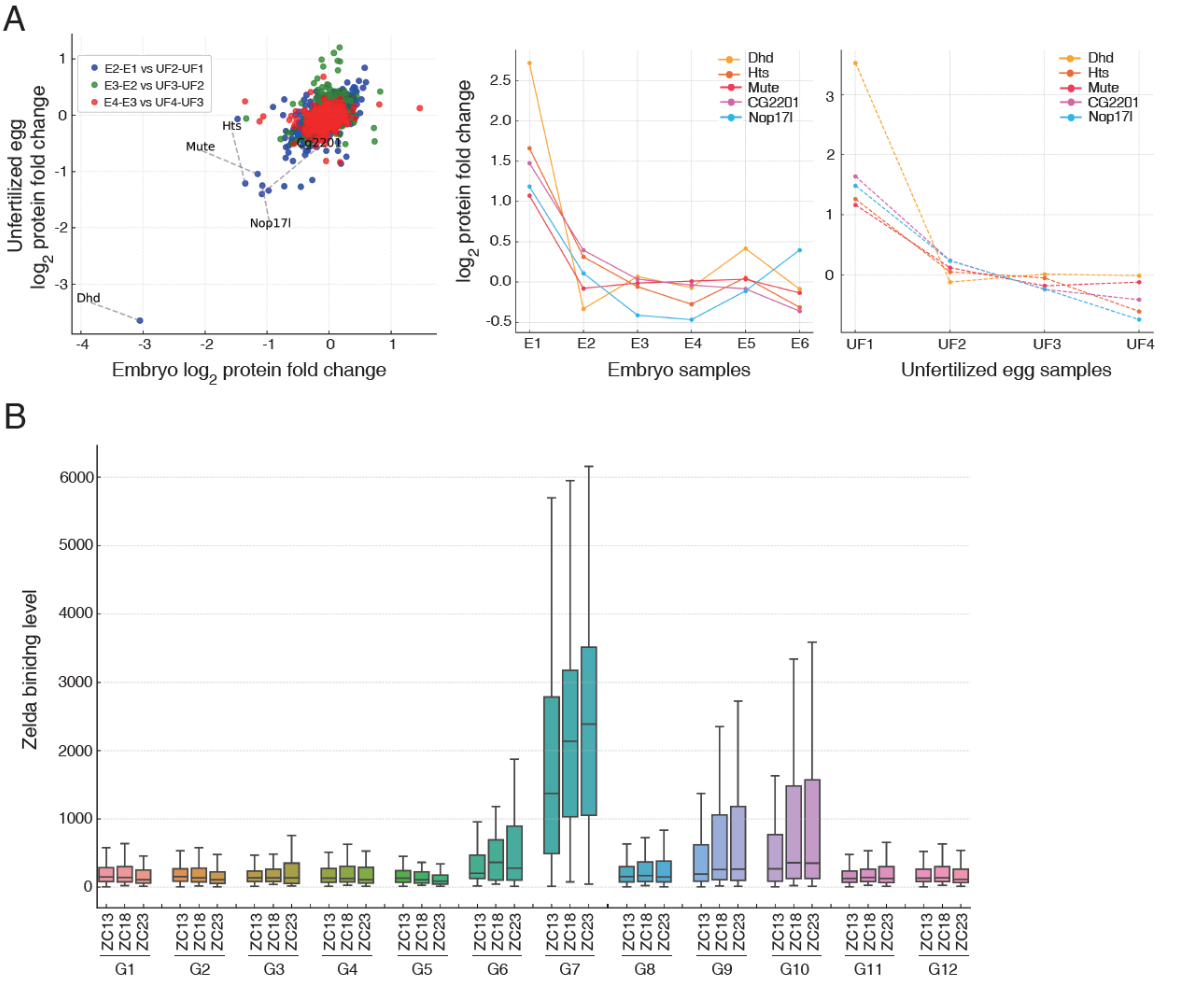

Figure S4. Dynamic changes in protein expression level based on specific regulatory mechanisms, Related to Figure 2

(A) Scatter plot showing protein expression level changes between developmental stages: E2 to E1 vs. UF2 to UF1, E4 to E3 vs. UF4 to UF3, and E3 to E2 vs. UF3 to UF2. Several proteins consistently downregulated at the earliest time point in both embryo and unfertilized egg samples are labeled in the plot (left panel). For highlighted proteins in the scatter plot, protein expression levels are shown across the embryo samples and unfertilized egg samples (right panels). (B) Box plot showing the k-means clustering group-specific distribution of Zld binding signal intensity at cell cycle 13 (ZC13), 18 (ZC18), and 23 (ZC23) of embryogenesis, as reported by Harrison et al. (2011)[52]. Each box represents the interquartile range (IQR) with the median value indicated by the line inside the box. Whiskers extend to the most extreme data points within 1.5 times the IQR.

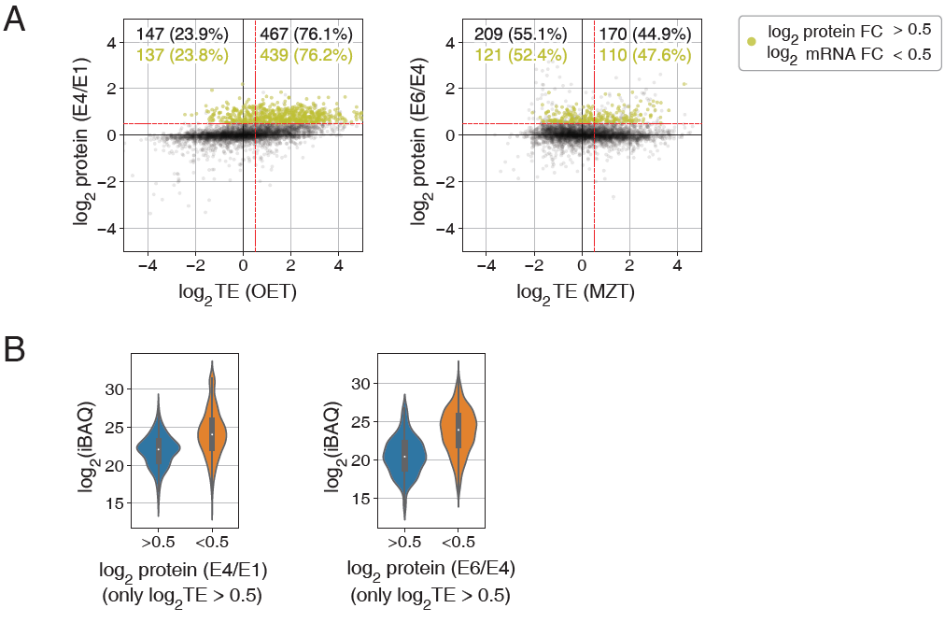

Figure S5. Relationship between protein expression changes and translational efficiency (TE) changes, Related to Figure 4

(A) Scatter plots depicting translation efficiency (TE) and protein level changes in *Drosophila* oocytes and embryos, based on previously published TE data[44]. The protein level changes from E1 to E4 and TE changes from oocyte stage 14 to 0–1 hr embryos (OET) are shown in the left panel. The protein level changes from E4 to E6 and TE changes from 0-1 hr to 3-4 hr AEL embryos (MZT) are shown in the right panel. The number and proportion of genes with increased protein expression (log2 fold change > 0.5) and corresponding TE changes (log2 fold change < 0.5 or > 0.5) are indicated. (B) Violin plots of log2 absolute protein quantities, grouped by protein fold changes (log2 fold change > 0.5 or < 0.5) in E1 to E4 (left) and E4 to E6 (right) respectively. Only proteins with TE > 0.5 are included in this plot.

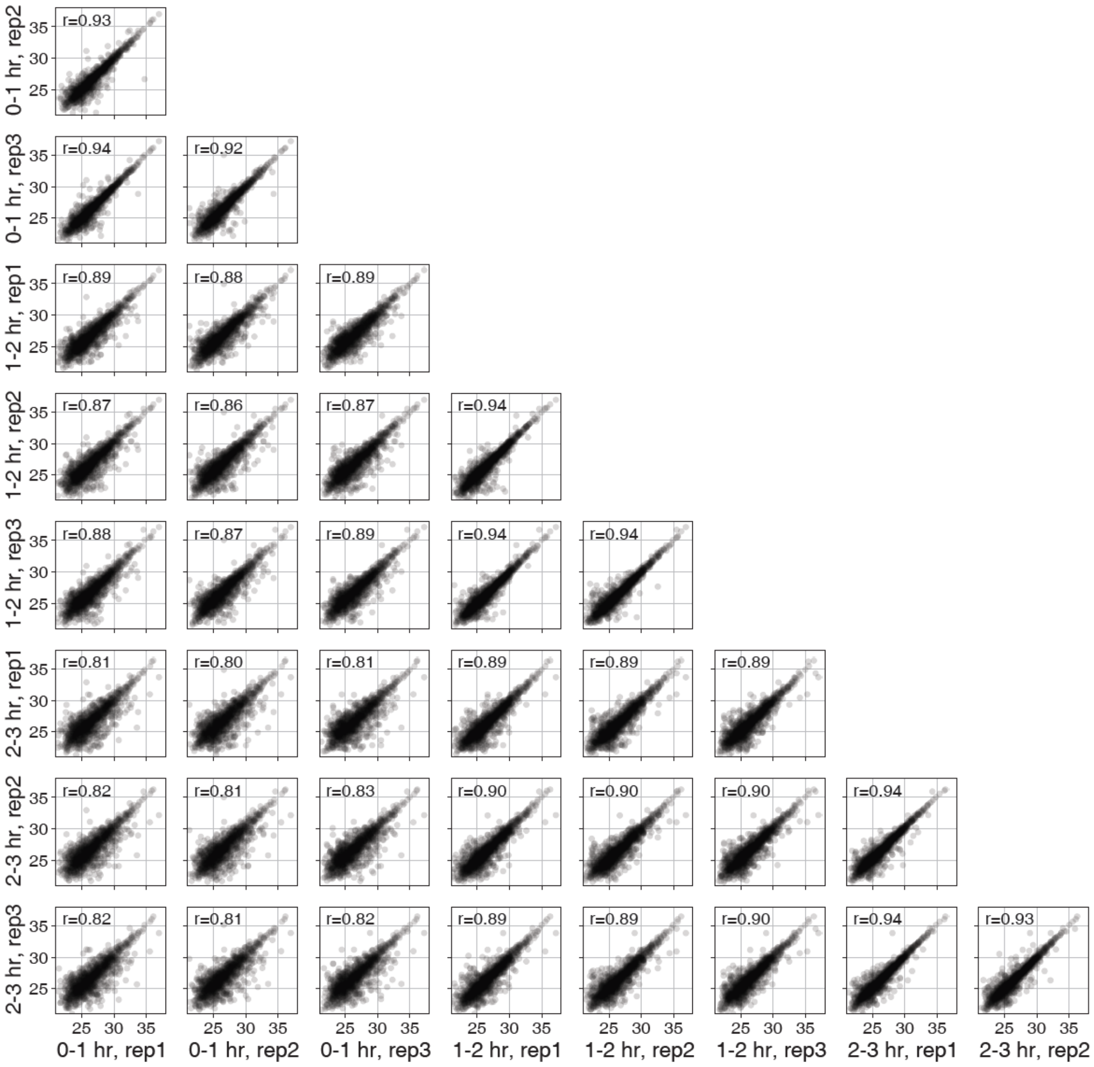

Figure S6. Reproducibility of the ubiquitome analysis, Related to Figure 5

Pairwise comparison of log2-transformed LFQ intensities for all ubiquitome samples. Pearson's correlation coefficients between each sample pair are shown in the plot.

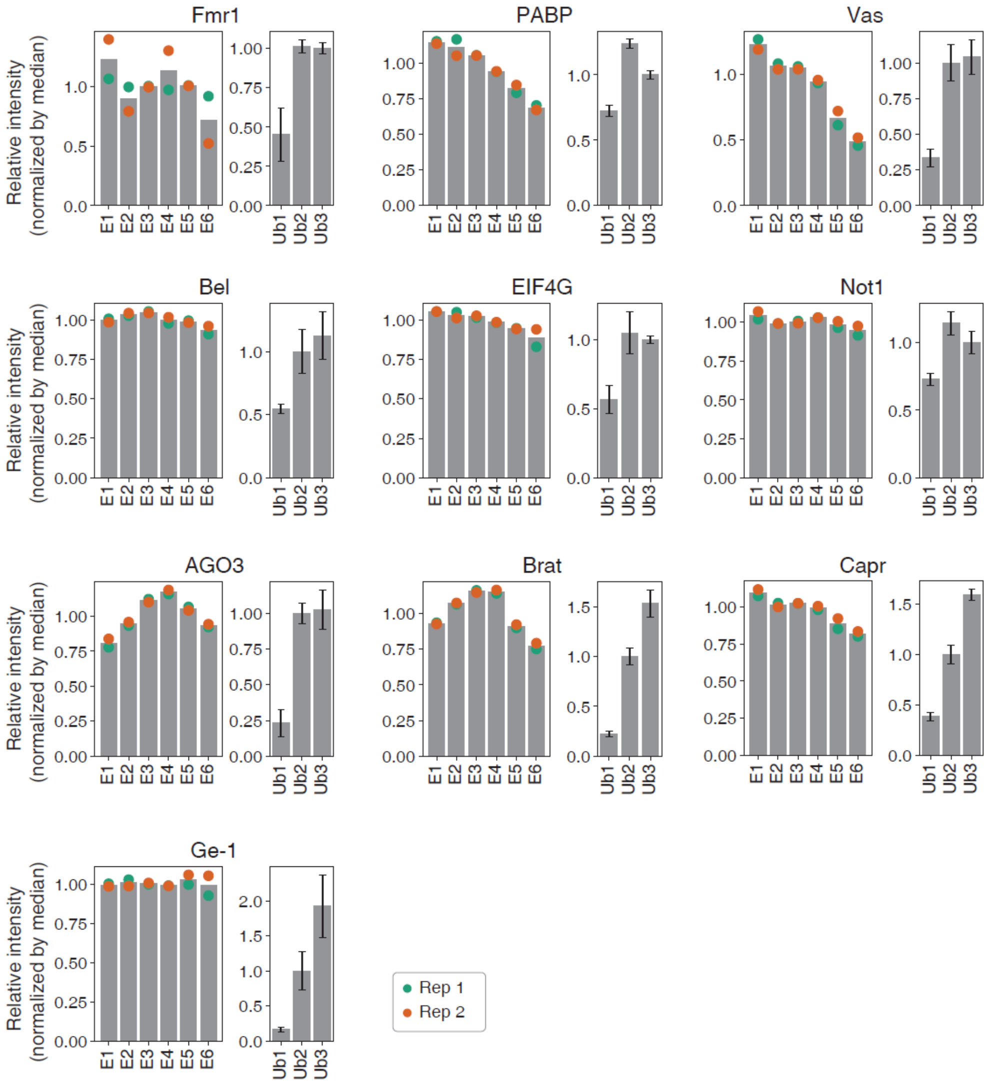

Figure S7. Representative ubiquitome proteins, Related to Figure 6

Total and ubiquitinated protein expression levels of selected genes are shown. The left bar plots show the change in mean total protein expression across time points. Green and orange dots indicate the total protein levels for individual replicates. The right bar plots represent the mean expression levels of ubiquitinated proteins. Error bars indicate the standard error of the mean.

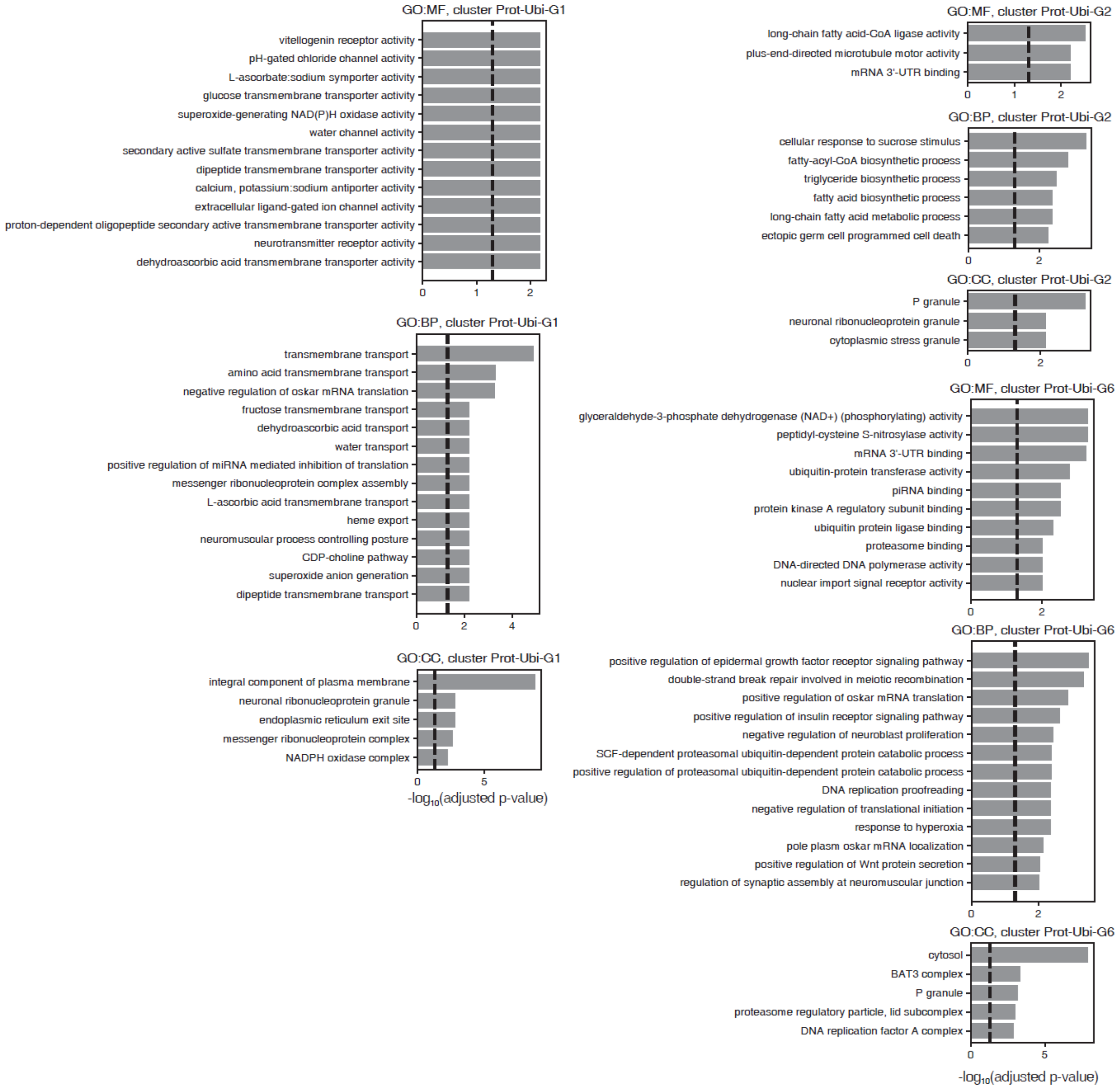

Figure S8. Overrepresented Gene Ontology (GO) terms for gene groups, clustered based on total and ubiquitinated protein expression levels, Related to Figure 6

GO terms are presented with enrichment p-values which were calculated and adjusted using the weight01 algorithm implemented in the TopGO R library. For the background group of the enrichment test, all proteins identified (FDR 1%, more than two unique peptides) and quantified in embryo samples (E1–E6) were included. Black dashed lines indicate p = 0.05. enriched GO terms were not defined for clusters G3, G4, and G7.

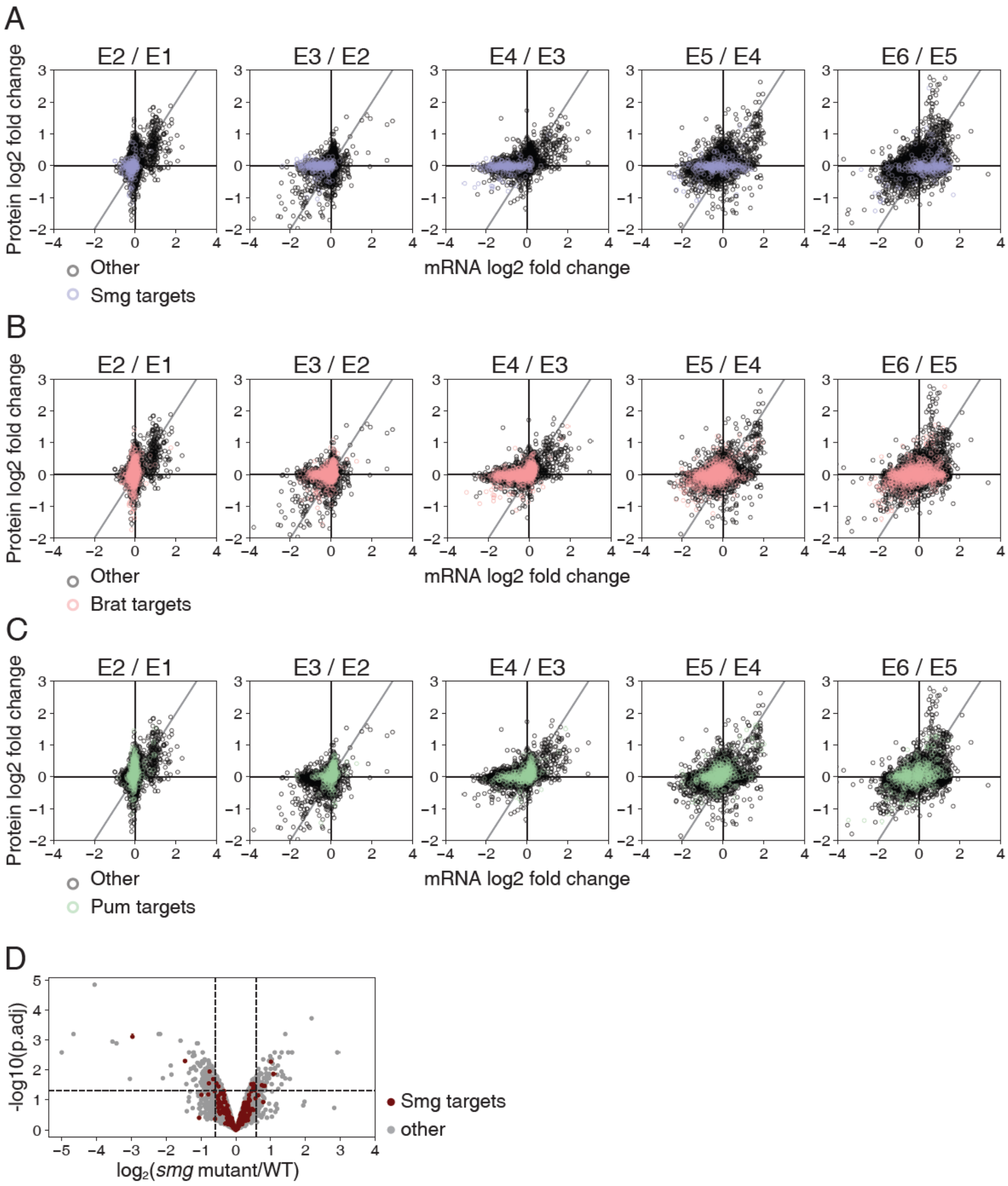

Figure S9. mRNA and protein expression level changes of RNA-binding proteins (RBPs) Smg, Brat, and Pum target genes , Related to Figure 7

(A–C) Fold changes in mRNA (x-axis) and protein (y-axis) expression across different embryo time points are shown. Genes targeted by the RBPs Smg (A, N = 308), Brat (B, N = 959), and Pum (C, N = 482) are highlighted in blue, red, and green dots, respectively, while black dots represent all other genes. Target lists were derived from Chen et al. (2014) for Smg and Laver et al. (2015) for Brat and Pum. (D) A volcano plot compares wild-type (WT) and *smg* mutant 0–2 hr AEL embryos, showing fold changes and adjusted p-values of differential expression. Protein quantities using TMT-6-plex labels were analyzed with the “limma R” package to calculate p-values, which were subsequently adjusted using the Benjamini-Hochberg method. Genes identified as Smg targets in Chen et al. (2014) were highlighted in red.

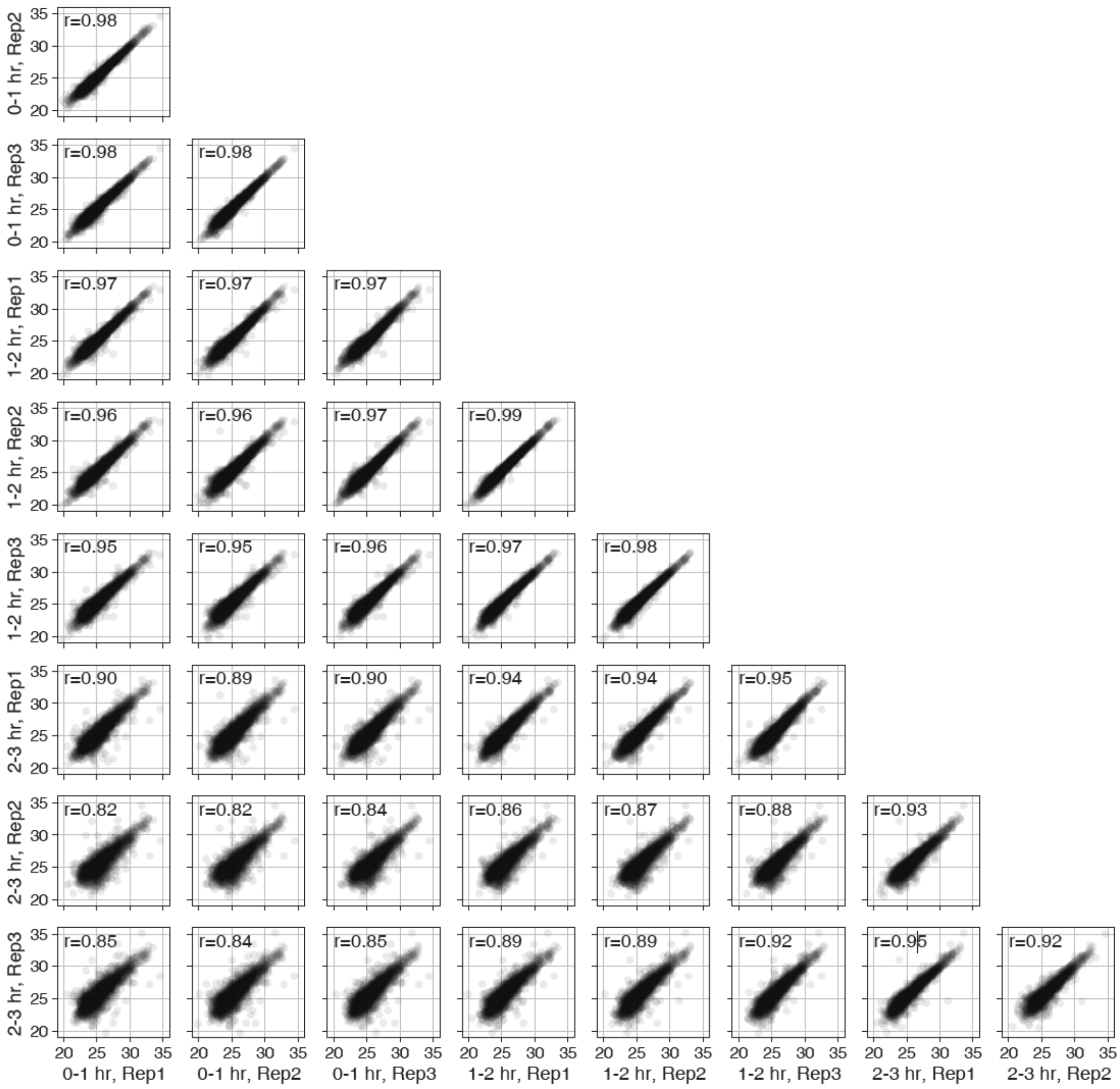

Figure S10. Pairwise comparisons of log2-transformed LFQ intensities for all FAX-RIC samples, Related to Figure 8

Pearson's correlation coefficients between each sample pair are shown in the plot.
