# Supplementary figures and images for: Gene expression dynamics before and after zygotic gene activation in Drosophila early embryogenesis
Source: iScience. 2025 Aug 7;28(9):113272. doi: 10.1016/j.isci.2025.113272 (PMC12396305; doi:10.1016/j.isci.2025.113272)

Figure 2D

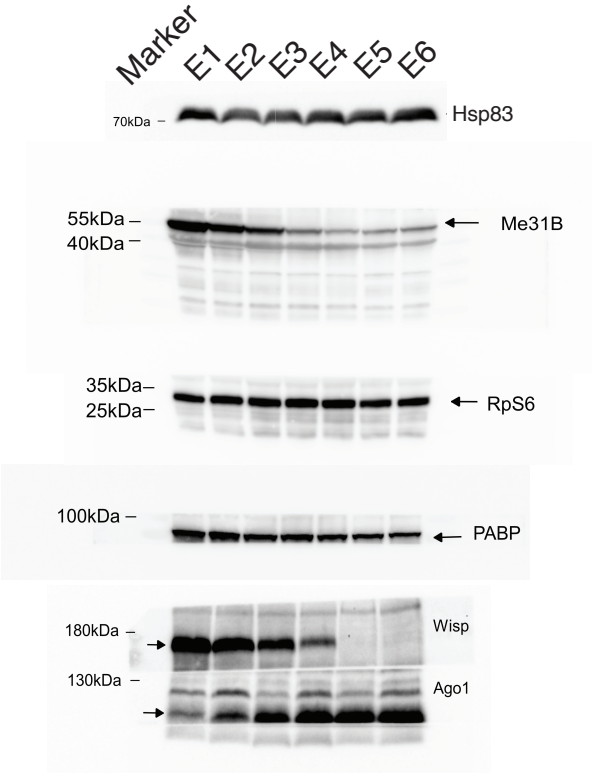

Supplement: Data S1. Original images for blots, related to Figure2D [file mmc2.pdf]
